# A qualitative transcriptional signature for the histological reclassification of lung squamous cell carcinomas and adenocarcinomas

**DOI:** 10.1186/s12864-019-6086-2

**Published:** 2019-11-21

**Authors:** Xin Li, Gengen Shi, Qingsong Chu, Wenbin Jiang, Yixin Liu, Sainan Zhang, Zheyang Zhang, Zixin Wei, Fei He, Zheng Guo, Lishuang Qi

**Affiliations:** 1 College of Bioinformatics Science and Technology, Harbin Medical University, Harbin, 150086 China; 2 Fujian Key Laboratory for Translational Research, Institute of Translational Medicine, Fujian Medical University, Fuzhou, 350001 China; 3 Department of Medical Oncology, Harbin Medical University Cancer hospital, Harbin, 150081 China; 4 Key Laboratory of Ministry of Education for Gastrointestinal Cancer, Department of Epidemiology and Health Statistics, School of Public Health, Fujian Medical University, Fuzhou, 350001 China; 5 Department of Bioinformatics, Key Laboratory of Ministry of Education for Gastrointestinal Cancer, School of Basic Medical Sciences, Fujian Medical University, Fuzhou, 350001 China; 6 Key laboratory of Medical Bioinformatics, Fujian Province, Fuzhou, 350001 China

**Keywords:** Non-small cell lung cancer, Histological subtype, Pathological assessment, Relative gene expression orderings, Qualitative transcriptional signature

## Abstract

**Background:**

Targeted therapy for non-small cell lung cancer is histology dependent. However, histological classification by routine pathological assessment with hematoxylin-eosin staining and immunostaining for poorly differentiated tumors, particularly those from small biopsies, is still challenging. Additionally, the effectiveness of immunomarkers is limited by technical inconsistencies of immunostaining and lack of standardization for staining interpretation.

**Results:**

Using gene expression profiles of pathologically-determined lung adenocarcinomas and squamous cell carcinomas, denoted as pADC and pSCC respectively, we developed a qualitative transcriptional signature, based on the within-sample relative gene expression orderings (REOs) of gene pairs, to distinguish ADC from SCC. The signature consists of two genes, *KRT5* and *AGR2*, which has the stable REO pattern of *KRT5* > *AGR2* in pSCC and *KRT5* < *AGR2* in pADC. In the two test datasets with relative unambiguous NSCLC types, the apparent accuracy of the signature were 94.44 and 98.41%, respectively. In the other integrated dataset for frozen tissues, the signature reclassified 4.22% of the 805 pADC patients as SCC and 12% of the 125 pSCC patients as ADC. Similar results were observed in the clinical challenging cases, including FFPE specimens, mixed tumors, small biopsy specimens and poorly differentiated specimens. The survival analyses showed that the pADC patients reclassified as SCC had significantly shorter overall survival than the signature-confirmed pADC patients (log-rank *p* = 0.0123, HR = 1.89), consisting with the knowledge that SCC patients suffer poor prognoses than ADC patients. The proliferative activity, subtype-specific marker genes and consensus clustering analyses also supported the correctness of our signature.

**Conclusions:**

The non-subjective qualitative REOs signature could effectively distinguish ADC from SCC, which would be an auxiliary test for the pathological assessment of the ambiguous cases.

## Background

Lung cancer is the most frequent cause of cancer-related deaths worldwide. Non-small cell lung cancer (NSCLC) represents around 80% of lung cancers [1], with two major histological subtypes: adenocarcinoma (ADC) and squamous cell carcinoma (SCC) [2]. Despite sharing many biological features, ADC and SCC differ in their cell of origin, location within the lung and tumor progression [1, 3], suggesting that they are distinct diseases that develop through differential molecular mechanisms. Consequently, some therapy regimens for NSCLC are histology dependent. For example, compared with SCC patients, ADC patients have a higher response rate to treatment of the epidermal growth factor receptor (EGFR) tyrosine kinase inhibitor [4–6]. The angiogenesis inhibitor bevacizumab is approved for non-squamous patients but forbidden to SCC patients due to the high rate of life-threatening pulmonary hemorrhag [4, 7]. Similarly, another chemotherapy drug, pemetrexed, also has been demonstrated efficacy for ADC or non-squamous patients [8]. These discrepancies in tumor biology and response to drug treatment highlight the importance to distinguish ADC from SCC accurately.

Microscopic morphological features observed from hematoxylin-eosin (HE) staining are currently the “golden” standard for the lung cancer histological classification. In general, if there is an adequate tumor specimen and the tumor is well or moderately differentiated, imaging technique is sufficient to determine ADC or SCC [1]. However, the histological classification for the poorly differentiated specimens or the small biopsy specimens, which account for about 70% of the initial lung cancer diagnoses [9], is still a challenge. Therefore, immunohistochemistry (IHC) detection of subtype-specific markers has been proposed for assisting the histological classification of NSCLC [10, 11]. Before the recommendations of the WHO 2015 Classification of lung cancer, most of the poorly differentiated NSCLC cases without morphologic evidence of glandular or squamous differentiation are assigned to the large cell carcinoma (LCC) subtype [12, 13]. However, Rekhtman et al. have reported that, except LCC with neuroendocrine features (LCNEC), most LCC should be classified as ADC or SCC [12]. Currently, many LCCs identified according to previous criteria can be reclassified as ADC or SCC subtype based on their immunomarkers [12, 13]. However, even with the auxiliary of immunomarkers, there is still a certain percentage of misclassified cases because of the subjective diagnoses of HE staining or immunostaining results by pathologists using varying pathological criteria or interpretations [14]. Additionally, based on the combinations of SCC and ADC immunomarkers, such as *TTF-1* and *p63* [11], there is still about 10% samples could not be classified as they are both positive or negative of two immunomarkers [15].

Therefore, in recent years, considerable efforts have been devoted to extracting signatures based on gene expression profiles to stratify ADC and SCC [1, 16]. However, most of the reported transcriptional signatures, such as the 42-gene signature [1], are based on risk scores summarized from the quantitative expression measurements of the signature genes, which lack robustness for clinical applications due to large measurement batch effects [17] and quality uncertainties of clinical samples [18–20].

Fortunately, the within-sample relative expression orderings (REOs) of genes, which are the qualitative transcriptional characteristics of samples, are robust against to experimental batch effects and disease signatures based on REOs can be directly applied to samples at the individualized level [21–26]. Besides, we have reported that the within-sample REOs of genes are highly robust against to partial RNA degradation during specimen storage and preparation [18], varied proportions of the tumor cells in tumor tissues [19], and low-input RNA specimens [20]. Therefore, it is worthwhile to apply the within-sample REOs to find a robust qualitative signature for distinguishing ADC from SCC.

In this study, we developed a REOs-based qualitative signature for individualized NSCLC histological reclassification. We tested the robustness of the signature in two datasets with relative unambiguous NSCLC types, concordantly determined by two independent routine pathologists. For the other test datasets, we performed the survival analyses, proliferative activity analyses, subtype-marker genes expressions and consensus clustering analyses to provide evidences that the signature could rectify some misclassifications of histological subtypes by routine pathological assessments. Especially, the sample reclassifications by the signature were validated in various specimen types, including the frozen tissue specimens, formalin fixed paraffin-embedded (FFPE) tissue specimens, small biopsy specimens, mixed tumor specimens with high varied proportions of tumor cells and poorly differentiated tumor (LCC) specimens. Therefore, this signature would be an effective auxiliary tool for precise diagnoses of lung SCC and ADC.

## Results

### Identification of the signature for distinguishing ADC from SCC

**Fig. 1:**
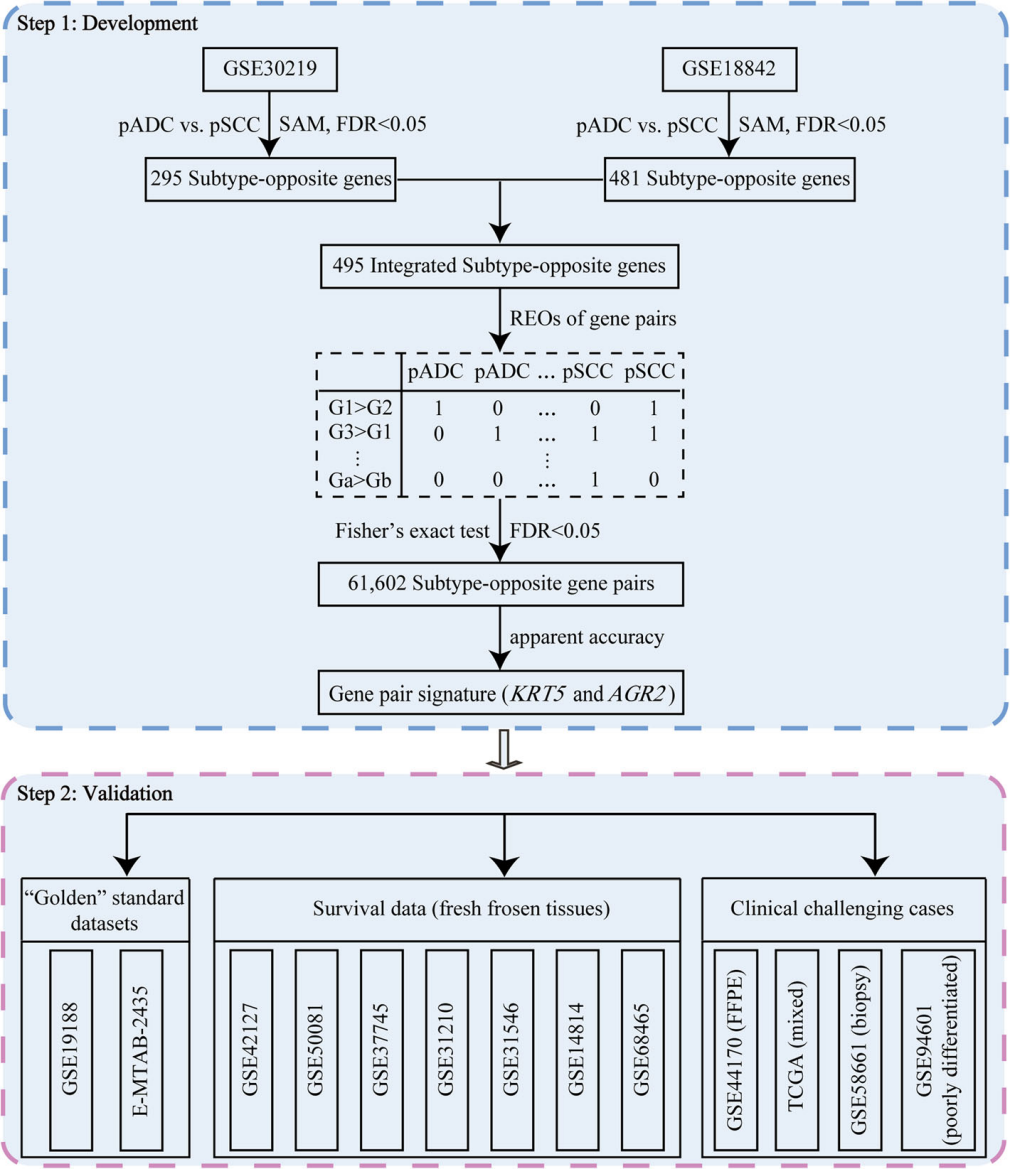
The flowchart of this study. Using gene expression profiles of pADC and pSCC, we developed a qualitative transcriptional signature to individually distinguish ADC from SCC. The signature was tested in “golden” standard dataset, fresh frozen samples with survival data and clinical challenging cases, including FFPE specimens, mixed tumors, small biopsy specimens and poorly differentiated specimens. The pADC and pSCC represent pathologically-determined squamous cell carcinoma and pathologically-determined adenocarcinoma, respectively

Figure 1 describes the flowchart of this study. First, from the 20,283 genes detected in the GSE30219 dataset (Table 1), we extracted 10,474 DE genes between the 85 pADC samples and the 14 normal controls, and 14,533 DE genes between the 61 pSCC samples and the 14 normal controls (SAM, FDR < 0.05). Interestingly, we found 295 genes that were DE genes in both the pADC and pSCC samples but with opposite dysregulated directions in the two types of samples when compared with the normal controls, and defined them as the subtype-opposite genes. Similarly, from the 20,283 genes detected in the GSE18842 dataset (Table 1), we extracted 9281 DE genes for the 14 pADC samples and 13,141 DE genes for the 31 pSCC samples when compared to the 45 normal controls (SAM, FDR < 0.05). And, 481 subtype-opposite genes were identified in this dataset. Notably, all the 148 overlapped subtype-opposite genes between the two datasets had consistently dysregulated directions in both pADC and pSCC samples, compared with the normal controls, respectively. Given that a dataset may usually capture only a part of all DE genes due to insufficient statistical power [27, 28], we integrated together the subtype-opposite genes extracted from the two datasets, excluding the 133 genes that were subtype-opposite genes in one dataset but had inconsistent dysregulation directions (without statistical control) in the other dataset. Finally, we obtained 495 subtype-opposite genes to develop the qualitative transcriptional signature for distinguishing ADC from SCC. Then, we utilized the subtype-opposite genes to develop a qualitative transcriptional signature for distinguishing ADC from SCC. In the training data integrated from two microarray datasets (GSE30219 and GSE18842), including 99 pADC samples and 92 pSCC samples, from 122,265 gene pairs consisting of the subtype-opposite genes, we extracted 61,602 gene pairs with potentially subtype-opposite REO patterns (E*a* > E*b* in pSCC or equally E*b* > E*a* in pADC) occurring significantly more frequently in pSCC samples than in pADC samples (Fisher’s exact test, FDR < 0.05). Next, for each subtype-opposite gene pair, we calculated the apparent accuracy of the gene pair for distinguishing ADC from SCC in the training data, as the pathological assessments are not 100% reliable [29]. Finally, using each of the top 50 subtype-opposite gene pairs (Additional file 1: Table S2) as a seed, we performed a forward selection procedure and obtained 50 optimal sets of gene pairs (see Methods), among which two sets reached the highest apparent accuracy (98.43%). One set contained only one gene pair, *KRT5* and *AGR2*, as the addition of any other gene pair did not increase the apparent accuracy. The other set, consisting of two gene pairs, also contained the gene pair (*KRT5* and *AGR2*), indicating that this gene pair had the optimal performance. Therefore, the gene pair, *KRT5* and *AGR2*, was selected as the signature for distinguishing ADC from SCC. The classification rule of the signature is that a sample was classified as SCC if the mRNA expression level of *KRT5* was higher than that of *AGR2*; otherwise ADC. According to the classification rule, two of the 61 pSCC samples in the GSE30219 dataset and one of the 31 pSCC samples in the GSE18842 dataset were reclassified as ADC and all the 99 pADC samples in the two datasets were confirmed by the signature.

**Table 1:** The datasets analyzed in this study

Types	Data Source	Database	Platform	pADC	pSCC	Normal
Train (frozen)	GSE30219	GEO	Affy. Plus 2	85	61	14
	GSE18842	GEO	Affy. Plus 2	14	31	45
Total	–	–	–	99	92	59
“Golden”standard data	GSE19188	GEO	Affy. Plus 2	45	27	–
	E-MTAB-2435	ArrayExpress	Affy. Plus 2	0	63	–
Total	–	–	–	45	90	–
Integrated data (frozen)	GSE42127^a^	GEO	Illu. WG V3.0	90	32	–
	GSE50081^a^	GEO	Affy. Plus 2	127	43	–
	GSE37745^a^	GEO	Affy. Plus 2	40	24	–
	GSE31210^a^	GEO	Affy. Plus 2	204	0	–
	GSE31546^a^	GEO	Affy. Plus 2	13	0	–
	GSE14814^a^	GEO	Affy. U133A	32	26	–
	GSE68465^a^	GEO	Affy. U133A	299	0	–
	Total	–	–	805	125	–
FFPE	GSE44170	GEO	Affy. U133A	0	38	–
Mixed	TCGA	TCGA	Illu. HiSeqV2	498	499	–
Biopsy	GSE58661	GEO	Affy. 2.0	42	36	–
Poorly differentiated	GSE94601	GEO	Illu. HT V4.0	19^b^	4^b^	–
Total	–	–	–	1364	702	–

*pADC* pathologically-determined ADC, *pSCC* pathologically-determined SCC, *Affy. Plus 2* Affymetrix Plus 2, *Affy. U133A* Affymetrix U133A, *Affy. 2.0* Rosetta/Merck Human RSTA Custom Affymetrix 2.0, *Illu. WG V3.0* Illumina HumanWG-6 V3.0, *Illu. HT V3.0* Illumina HumanHT-12 V3.0, *Illu. HiSeqV2* Illumina HiSeqV2, *Illumina HT V4.0* Illumina HumanHT-12 V4.0

^a^the data records the survival information of patients treated with curative surgery resection only

^b^the 19 pADCs and 4 pSCCs samples are poorly differentiated which were improperly assigned to LCC subtype before and reclassified by the authors using ADC and SCC immunomarkers

### *Krt5* and *Agr2* proteins immunostaining in pADC and pSCC

**Fig. 2:**
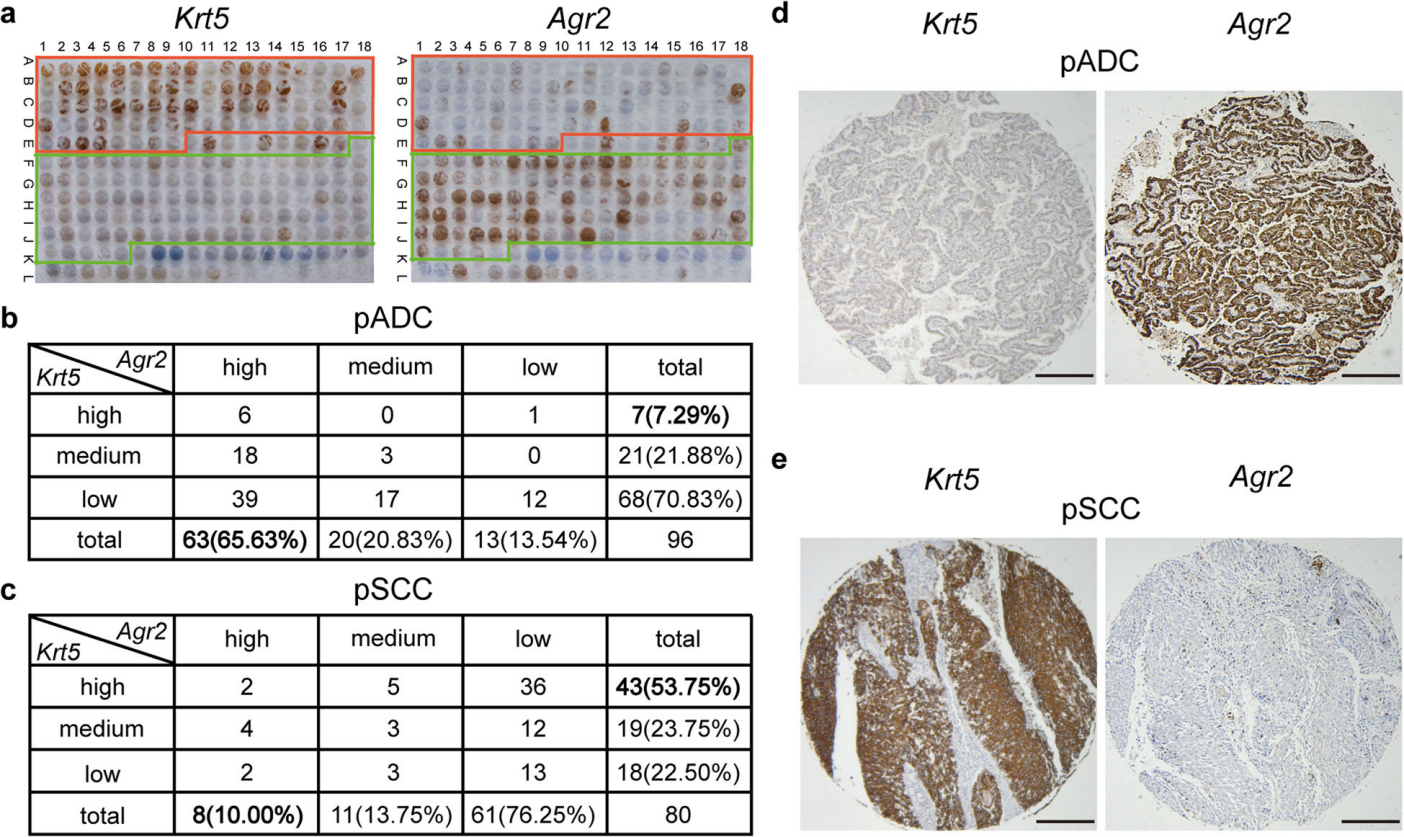
Immunohistochemical analysis of *Krt5* protein and *Agr2* protein expressions in human lung cancer tissue microarray. **a** *Krt5* and *Agr2* proteins expression profile in lung cancer tissue array. The red frame containing samples from A1-E8 are pSCC. The green frame containing samples from E18-K5 are pADC. The remaining samples are the other subtypes of lung cancer and normal controls. **b**, **c** Inverse correlation between *Krt5* protein and *Agr2* protein expressions in pADC (**b**) and pSCC (**c**) samples. The protein expression score was quantified and considered as low, medium and high expression, basing on a multiplicative index of the average staining intensity and the extent of staining (see Methods). **d**, **e** Representative immunohistochemical staining results of *Krt5* and *Agr2* proteins in pADC (**d**) and pSCC (**e**) samples. Scale bar, 1 mm

Immunohistochemical analysis of the *Krt5* and *Agr2* proteins was performed for 96 pADC samples and 80 pSCC samples, derived from Anenabio, Xi’an, China. The IHC results for *Krt5* and *Agr2* proteins are shown in Fig. 2a. For the 96 pADC samples, *Agr2* protein was highly expressed in 63 (65.63%) samples, while *Krt5* protein was only highly expressed in 7 (7.29%) samples (Fig. 2b). In contrary, for the 80 pSCC samples, *Krt5* protein was highly expressed in 43 (53.75%) samples, while *Agr2* protein was only highly expressed in 8 (10.00%) samples (Fig. 2c). The results suggested that *Krt5* protein was mainly expressed in pSCC samples, while *Agr2* protein was mainly expressed in pADC samples. The representative IHC staining of *Krt5* and *Agr2* proteins in pADC and pSCC samples are represented in Fig. 2d and e, respectively. The results provided the biological evidences of the signature in distinguishing ADC from SCC. However, the IHC analysis also showed that 6 (6.25%) pADC samples and 2 (2.50%) pSCC were highly expressed of both *Krt5* and *Agr2* proteins, and 12 (12.50%) pADC and 13 (16.25%) pSCC samples were low expressed of both *Krt5* and *Agr2* proteins, suggesting the limitation of IHC of immunomarkers in distinguishing ADC from SCC.

### Validation of the signature

First, we tested the signature on two datasets (GSE19188 and E-MTAB-2435) with relative unambiguous NSCLC types, which were concordantly determined by two independent routine pathologists. In the GSE19188 dataset with 45 pADC and 27 pSCC samples, the apparent accuracy of the signature for pADC (sensitivity) was 93.33%, the apparent accuracy for pSCC (specificity) was 96.30%, and the overall apparent accuracy was 94.44% (Table 2). Similar, in the E-MTAB-2435 dataset, the apparent accuracy of the signature for 63 pSCC samples (specificity) was 98.41% (Table 2). Additionally, in the two test datasets, we also compared our signature with the other 49 optimal sets of gene pairs obtained from the training data, and found our signature (*KRT5* and *AGR2*) had the optimal performance (Additional file 1: Table S2), suggesting the robustness of our signature in distinguishing ADC from SCC.

**Table 2:** The performance of our signature for pSCC and pADC samples in test datasets

	Data Source	pADC	pSCC	A- Sen (rate)	A- Spe (rate)	A- Acc (rate)	Re (SCC) (rate)	Re (ADC) (rate)
“Golden”standard data	GSE19188	45	27	93.33%	96.30%	94.44%	3 (6.67%)	1 (3.70%)
	E-MTAB-2435	0	63	–	98.41%	98.41%	–	1 (1.59%)
Total	–	45	90	93.33%	97.78%	96.30%	3 (6.67%)	2 (2.22%)
Integrated data (frozen)	GSE42127	90	32	90.00%	84.38%	88.52%	9 (10.00%)	5 (15.62%)
	GSE50081	127	43	88.19%	86.05%	87.65%	15 (11.81%)	6 (13.95%)
	GSE37745	40	24	95.00%	87.50%	92.19%	2 (5.00%)	3 (12.50%)
	GSE31210	204	0	99.02%	–	99.02%	2 (0.98%)	–
	GSE31546	13	0	100%	–	100%	0 (0.00%)	–
	GSE14814	32	26	93.75%	96.15%	94.83%	2 (6.25%)	1 (3.85%)
	GSE68465	299	0	98.66%	–	98.66%	4 (1.34%)	–
	Total	805	125	95.78%	88.00%	94.73%	34 (4.22%)	15 (12.00%)
FFPE	GSE44170	0	38	–	92.11%	92.11%	–	3 (7.89%)
Mixed	TCGA	498	499	97.59%	83.57%	90.75%	12 (2.41%)	82 (16.43%)
Biopsy	GSE58661	42	36	95.24%	88.89%	92.31%	2 (4.76%)	4 (11.11%)
Poorly differentiated	GSE94601	19^a^	4^a^	100%	50.00%	91.30%	0 (0.00%)	2 (50.00%)
Total	–	1364	702	96.48%	84.90%	92.55%	48 (3.52%)	106 (15.10%)

A-Sen represents the apparent sensibility, A-Spe represents the apparent specificity and A-acc represents the apparent accuracy

Re (SCC) represents the number of pADC samples reclassified as SCC by signature

Re (ADC) represents the number of pSCC samples reclassified as ADC by signature

^a^the 19 pADCs and 4 pSCCs samples are poorly differentiated which were improperly assigned to LCC subtype before and reclassified by the authors using ADC and SCC immunomarkers

**Fig. 3:**
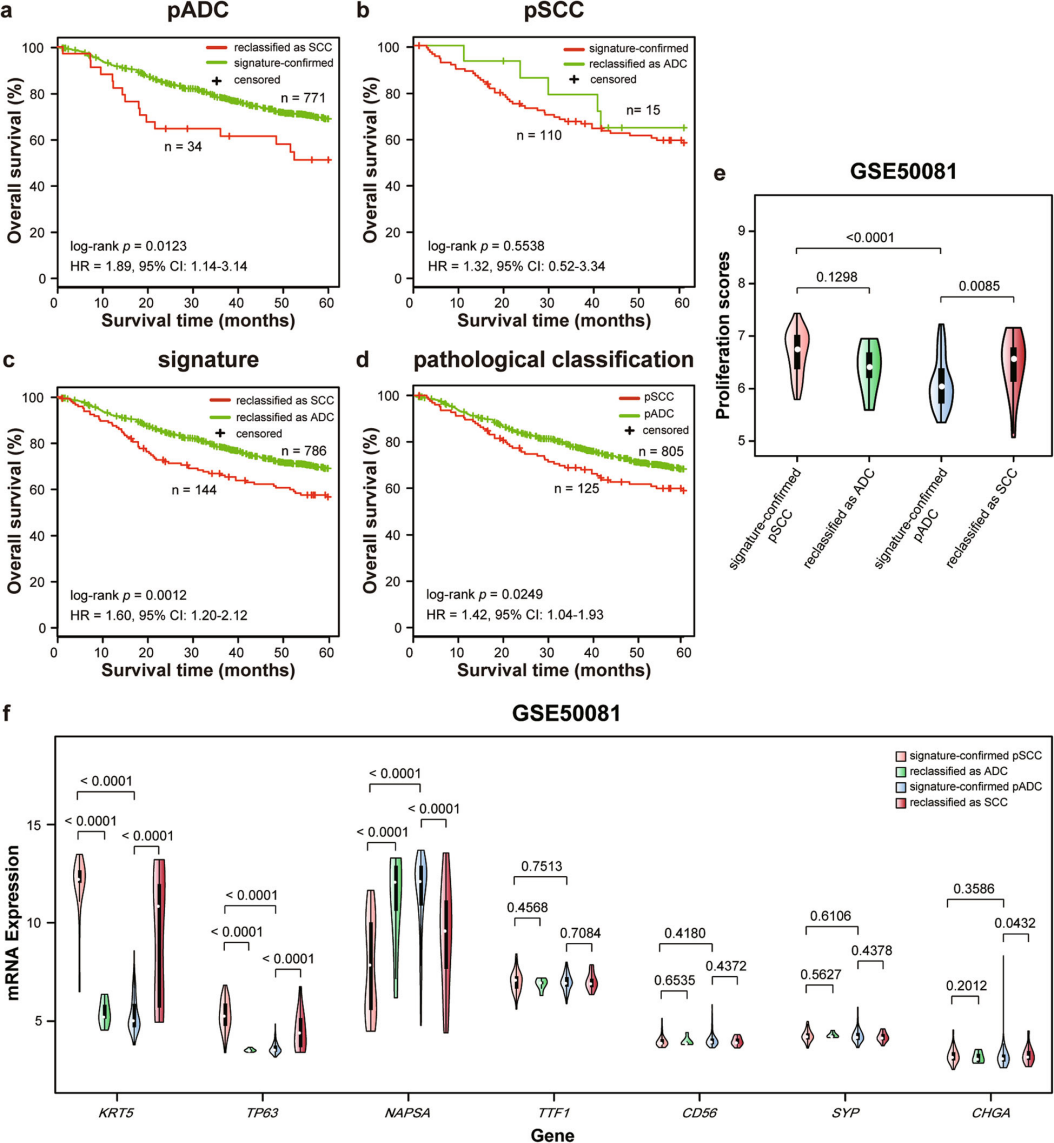
The validation of the reclassifications by the signature for fresh frozen samples with survival data. **a** Kaplan-Meier curves of overall survival (OS) respectively for the pADC reclassified as SCC and the signature-confirmed pADC groups. **b** Kaplan-Meier curves of OS respectively for the pSCC reclassified as ADC and the signature-confirmed pSCC groups. **c**, **d** Kaplan-Meier curves of OS respectively for the SCC and ADC groups reclassified by the signature (**c**) and original pathological assessment (**d**). **e** The violin plot of proliferation scores in the reclassified and signature-confirmed samples, respectively, in the GSE50081 dataset with the higher reclassification rate in the fresh frozen samples. Wilcoxon rank sum test was used to test the difference of proliferation scores between two groups. **f** The violin plot of mRNA expressions of the seven subtype-specific marker genes in the GSE50081 dataset. The subtype-specific marker genes include ADC marker genes (*NAPSA*, *TTF1*), SCC marker genes (*KRT5*, *TP63*) and neuroendocrine marker genes (*CD56*, *SYP*, *CHGA*). The RankProd (RP) algorithm was used to test the difference of the subtype-specific marker genes between reclassified samples and signature-confirmed samples

Since the histological classification of NSCLC in the other test datasets were not mentioned whether they were confirmed by independent pathologists or performed additional detection, we calculated the apparent accuracy of the signature and performed several biological analyses to indirectly support the reclassification of our signature. Firstly, based on the knowledge that SCC patients suffer poorer prognoses than ADC patients [30], we evaluated the correctness of the reclassification by our signature through survival analyses. For this purpose, we integrated 7 datasets recording survival information of patients treated with curative surgery resection only, including 805 pADC samples and 125 pSCC samples. In the integrated dataset, the apparent sensitivity (pADC prediction) of the signature was 95.78% and the apparent specificity (pSCC prediction) was 88.00% (Table 2). Notably, the signature reclassified a total 34 (4.22%) pADC samples as SCC and a total 15 (12.00%) pSCC samples as ADC. The survival analyses showed that the 34 pADC patients reclassified as SCC had significantly shorter OS than the remained 771 signature-confirmed pADC patients (log-rank *p* = 0.0123, HR = 1.89, 95% CI = 1.14–3.14, Fig. 3a), whereas the 15 pSCC patients reclassified as ADC showed longer OS than the 110 signature-confirmed pSCC patients but without significantly difference (log-rank *p* = 0.5538, HR = 1.32, 95% CI = 0.52–3.34, Fig. 3b). Multivariate Cox analysis showed that the pADC patients reclassified as SCC also had significantly shorter OS than the signature-confirmed pADC patients (*p* = 0.0458, HR = 1.72, 95% CI = 1.01–2.93, Table 3), after adjusting for data centers and clinical parameters, including stage, age and gender. The multivariate results for data centers and clinical parameters are displayed in Table 3. Notably, the 144 SCC patients classified by our signature had significantly shorter OS than the 786 ADC patients classified by the signature (log-rank *p* = 0.0012, HR = 1.60, 95% CI = 1.20–2.12, Fig. 3c), which was more significant than the OS difference between the original pSCC and pADC groups (log-rank *p* = 0.0249, HR = 1.42, 95% CI = 1.04–1.93, Fig. 3d). The OS between the two histological subtypes classified by our signature remained significantly different (*p* = 0.0500, HR = 1.36, 95% CI = 1.00–1.85, Table 4) after adjusting for data centers and clinical parameters. Furthermore, in order to reduce the potential bias due to integration and truncation of survival time, we removed one dataset from the integrated data in turn and performed the survival analyses for each new integrated data. All the results showed that the OS differences between the two histological groups classified by our signature were more significant than that between the original histological groups (Additional file 1: Figure S1). The above results suggested that the signature could rectify some misclassifications by routine pathological assessment which confused the survival difference between the two histological subtypes. Besides, in the GSE50081 dataset with the highest reclassification rate (12.35%) in the integrated dataset, we analyzed the proliferative activities of the reclassified samples by calculating their proliferation scores. The results showed that the 15 pADC samples reclassified as SCC had significantly higher proliferation scores than the signature-confirmed pADC samples (Wilcoxon rank sum test, *p* = 0.0085, Fig. 3e), indicating that the pADC samples reclassified as SCC are more proliferative than the signature-confirmed pADC samples which may cause worse prognoses. While the 6 pSCC samples reclassified as ADC had lower proliferation scores than the signature-confirmed pSCC samples though the difference was not significant possibly due to the small sample size (Wilcoxon rank sum test, *p* = 0.1298, Fig. 3e). Next, we also performed differential expression analyses for the subtype-specific marker genes using the RankProd (RP) algorithm. The result showed that the 15 pADC samples reclassified as SCC had significantly increased mRNA expression of the two SCC marker genes (RP algorithm, *KRT5*: *p* < 0.0001; *TP63*: *p* < 0.0001, Fig. 3f), and decreased mRNA expressions of an ADC marker gene (RP algorithm, *NAPSA*: *p* < 0.0001, Fig. 3f) than the signature-confirmed pADC samples. In contrast, the 6 pSCC samples reclassified as ADC had significantly increased mRNA expression of an ADC marker gene (RP algorithm, *NAPSA*: *p* < 0.0001, Fig. 3f), and decreased mRNA expressions of the two SCC marker genes (RP algorithm, *KRT5*: *p* < 0.0001; *TP63*: *p* < 0.0001, Fig. 3f), respectively, when compared with the signature-confirmed pSCC samples. The mRNA expressions of three neuroendocrine marker genes were in very low level in all the samples. Finally, based on the mRNA expression measurements of top 1000 most variable genes in the GSE50081 dataset, the samples were optimally classified into two subgroups (*k* = 2) using consensus clustering (Additional file 1: Figure S2). The clustering result showed that 10 of the 15 pADC samples reclassified as SCC were clustered with the signature-confirmed SCC samples and 5 of the 6 pSCC samples reclassified as ADC were clustered with the signature-confirmed ADC samples (Additional file 1: Figure S2). Similar clustering results were observed for top 2000 and 3000 most variable genes (Additional file 1: Figure S3 and S4).

**Table 3:** Multivariate Cox regression analysis for the pADC reclassified as SCC samples in the integrated dataset

Variable	Hazard ratio	*p*	95% CI
Histological classification by the signature (reclassified as SCC vs. signature-confirmed ADC)	1.72	0.0458	1.01–2.93
Data centers	1.05	0.0887	0.99–1.12
Stage (III vs. II vs. I)	2.32	< 0.0001	1.95–2.76
Age (> 65 vs. ≤65)	1.56	0.0009	1.20–2.04
Gender (Male vs. Female)	1.54	0.0013	1.18–2.01

*CI* confidence interval

**Table 4:** Multivariate Cox regression analysis for the histological classification by the signature in the integrated dataset

Variable	Hazard ratio	*P*	95% CI
Histological classification by the signature (SCC vs. ADC)	1.36	0.0500	1.00–1.85
Data centers	1.05	0.0729	1.00–1.11
Stage (III vs. II vs. I)	2.08	< 0.0001	1.78–2.45
Age (> 65 vs. **≤**65)	1.54	0.0004	1.21–1.97
Gender (Male vs. Female)	1.54	0.0005	1.21–1.97

*CI* confidence interval

**Fig. 4:**
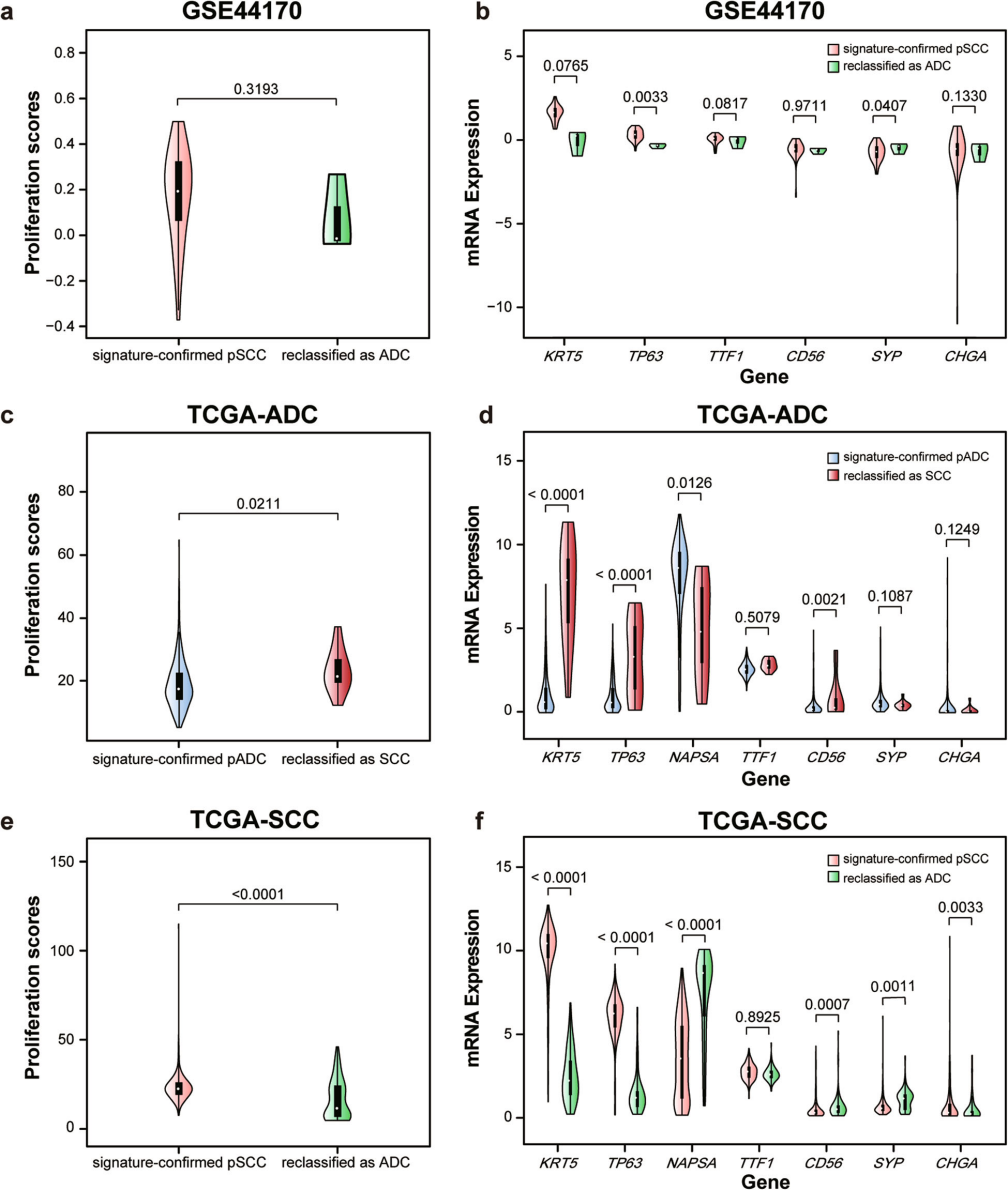
The validation of the reclassifications by the signature for the FFPE and mixed tumor specimens. **a** The violin plot of proliferation scores and **b** mRNA expressions of the subtype-specific marker genes in the GSE44170 dataset derived from FFPE specimens. **c** The violin plot of proliferation scores and **d** mRNA expressions of the subtype-specific marker genes in the TCGA-ADC dataset derived from mixed tumor specimens. **e** The violin plot of proliferation scores and **f** mRNA expressions of the subtype-specific marker genes in the TCGA-SCC dataset derived from mixed tumor specimens

Our previous study has demonstrated that REOs of gene pairs were highly stable in FFPE specimens with partial RNA degradation [18]. Here, we applied the signature to the GSE44170 dataset derived from FFPE specimens, and found the apparent accuracy of the signature for 38 pSCC samples (specificity) was 92.11% (Table 2). The 3 (7.89%) reclassified as ADC samples had lower proliferation scores than the signature-confirmed pSCC samples though the difference was not significant possibly due to the small sample size (Wilcoxon rank sum test, *p* = 0.3193, Fig. 4a). Moreover, the 3 reclassified samples had (marginally) significantly decreased mRNA expression of *KRT5* (RP algorithm, *p* = 0.0765, Fig. 4b) and *TP63* (RP algorithm, *p* = 0.0033, Fig. 4b), respectively, than the signature-confirmed pSCC samples. Consensus clustering was not performed as the dataset contains one subtype.

In another research, we also demonstrated that the REOs of gene pairs were robust against tumor cell variations in mixed tumor specimens [19]. Thus, we applied the signature to mixed tumor samples with 10% ~ 100% tumor cells in the TCGA datasets. In the TCGA-ADC dataset, the apparent accuracy of the signature for 498 pADC samples (sensitivity) was 97.59% (Table 2), which were not significantly related with tumor cell proportions (Spearman’s rank correlation, *p* = 0.9331). Here, the signature reclassified 12 (2.41%) pADC samples as SCC, whose proliferation scores were significantly higher than that of the signature-confirmed pADC samples (Wilcoxon rank sum test, *p* = 0.0211, Fig. 4c). And, these reclassified samples had significantly increased mRNA expressions of *KRT5* (RP algorithm, *p* < 0.0001, Fig. 4d) and *TP63* (RP algorithm, *p* < 0.0001, Fig. 4d), and decreased mRNA expression of *NAPSA* (RP algorithm, *p* = 0.0126, Fig. 4d), respectively, when compared with the signature-confirmed pADC samples. Similarly, in the TCGA-SCC dataset, the apparent accuracy of the signature for the 499 pSCC samples (specificity) was 83.57%, which were also not significantly related with tumor cell proportions (Spearman’s rank correlation, *p* = 0.8886). The 82 pSCC samples were reclassified as ADC by the signature and their proliferation scores were significantly lower than the signature-confirmed pSCC samples (Wilcoxon rank sum test, *p* < 0.0001, Fig. 4e). Comparing with the signature-confirmed pSCC samples, the 82 reclassified samples had significantly increased mRNA expression of *NAPSA* (RP algorithm, *p* < 0.0001, Fig. 4f) and decreased mRNA expressions of *KRT5* (RP algorithm, *p* < 0.0001, Fig. 4f) and *TP63* (RP algorithm, *p* < 0.0001, Fig. 4f), respectively.

**Fig. 5:**
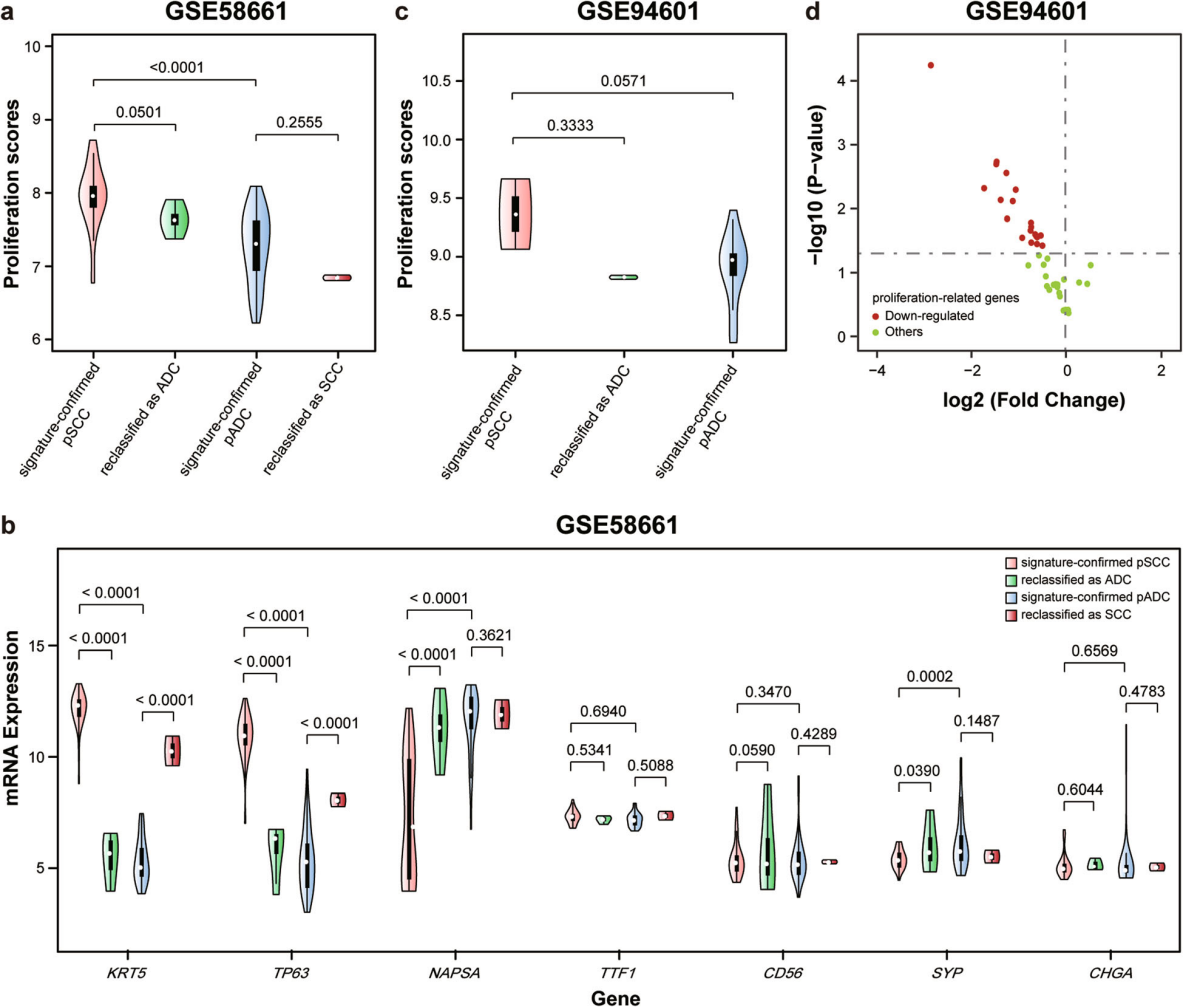
The validation of the reclassifications by the signature for small biopsy and poorly differentiated specimens. **a** The violin plot of proliferation scores and **b** mRNA expressions of the subtype-specific marker genes in the GSE58661 dataset derived from small biopsy specimens. **c** The violin plot of proliferation scores of 23 poorly differentiated specimens in the GSE94601 dataset. **d** The volcano plot of the differential expressions of the 44 proliferation-related genes in the pSCC samples reclassified as ADC when compared with the signature-confirmed pSCC samples. For the 44 proliferation-related genes, 20 genes were significantly differentially expressed and all the genes were down-regulated in the reclassified pSCC

Previously, we have reported that the REOs of gene pairs were also robust to low-input RNA specimens. Thus, we next tested the performance of the signature in the GSE58661 dataset for small biopsy specimens, including 42 pADC and 36 pSCC samples. The results that the apparent sensitivity (pADC prediction) and specificity (pSCC prediction) were 95.24 and 88.89%, respectively (Table 2). The 4 (11.11%) pSCC samples reclassified as ADC had marginally lower proliferation scores than the signature-confirmed pSCC (Wilcoxon rank sum test, *p* = 0.0501, Fig. 5a). Although the 2 pADC samples reclassified as SCC had lower proliferation scores, they had significantly increased mRNA expression of *KRT5* (RP algorithm, *p* < 0.0001, Fig. 5b) and *TP63* (RP algorithm, *p* < 0.0001, Fig. 5b) than the signature-confirmed pADC samples, indicating the correctness of the reclassification for the 2 samples. In contrast, the 4 pSCC samples reclassified as ADC had significantly increased mRNA expression of *NAPSA* (RP algorithm, *p* < 0.0001, Fig. 5b), and decreased mRNA expressions of *KRT5* (RP algorithm, *p* < 0.0001, Fig. 5b) and *TP63* (RP algorithm, *p* < 0.0001, Fig. 5b), than the signature-confirmed pSCC samples. Additionally, consensus clustering based on the top 1000 most variable genes showed that all the 4 pSCC samples reclassified as ADC by the signature were clustered with the signature-confirmed ADC samples (Additional file 1: Figure S5). Similar clustering results were observed for top 2000 and 3000 most variable genes (Additional file 1: Figure S6 and S7).

Finally, we also evaluated the performance of the signature for poorly differentiated specimens. In the GSE94601 dataset, the 19 and 4 poorly differentially samples initially assigned to the LCC subtype were reclassified to pADC and pSCC with the positive expressions of ADC markers (*NAPSA*/*TTF1*) and SCC markers (*Krt5*/*P40*), respectively. The apparent sensitivity (pADC prediction) and specificity (pSCC prediction) of the signature for poorly differentially samples were 100 and 50%, respectively (Table 2). The proliferation scores of the two pSCC reclassified as ADC samples were 8.82 and 8.83, respectively, which were similar with the median proliferation scores (8.89) of the 19 signature-confirmed pADC samples but lower than that (9.67 and 9.06) of the two signature-confirmed pSCC samples though the difference was not significant possibly due to the small sample size (Wilcoxon rank sum test, *p* = 0.3333, Fig. 5c). Additionally, we also performed differential expression analysis for the 44 proliferation-related genes [31] and found 20 genes that were differentially expressed in the 2 reclassified samples (RP algorithm, *p* < 0.05), and all the 20 genes were down-regulated (Fig. 5d), when compared with the two signature-confirmed pSCC samples. The results provide tentative evidences for the correctness of the reclassification by our signature. Consensus clustering was not performed as the small sample size of pSCC.

Taken together, the above results indicated that the signature could accurately distinguish ADC from SCC, especially for the clinical challenging cases.

## Discussion

In this study, we developed a robust qualitative transcriptional signature consisting of two genes (*KRT5* and *AGR2*), which can accurately distinguish ADC from SCC and is independent of the subjective experiences of pathologists. It is known that *Krt5* protein is expressed primarily in basal keratinocytes in the epidermis, whose overexpression is the unique characteristic of SCC [32]. Another gene in the signature, *Agr2*, is known as an adenocarcinoma antigen, which promotes cell migration and metastasis [33, 34]. Our immunohistochemistry results demonstrated that *krt5* protein was preferentially expressed in pSCC, while *Agr2* protein was preferentially expressed in pADC, providing biological evidences of the signature in distinguishing ADC from SCC. However, it is note that, there were 8 (4.55%) samples with high expressions of the two proteins and 25(14.20%) samples with low expressions of the two proteins, indicating that a certain percentage of patients could not be accurately classified by the immunohistochemical assessment. The limitation was also observed in the commonly used immunomarkers for ADC and SCC [15].

In the two test datasets with relative unambiguous NSCLC types, the apparent accuracy of the signature were 94.44 and 98.41%, respectively, suggesting the robustness of our signature in distinguishing ADC from SCC. In the other test datasets, including 2066 samples derived from frozen tissues and clinical challenging specimens, the apparent sensitivity (ADC prediction) was 96.48%, the apparent specificity (SCC prediction) was 84.90%, and the overall apparent accuracy was 92.55%. Notably, there were large differences in the apparent accuracy (or reclassification) rates among cohorts. It has been reported that different pathologists had discordant histological classifications for about one third of the cases because of their subjective diagnoses using varying criteria or interpretations [14], which might be the major factor causing different misclassification rates among cohorts. Therefore, we first supported the reclassifications of our signature by the survival and proliferative activity analyses based on the knowledge that SCC suffers more proliferative activity than ADC, which may cause worse prognoses [30, 35]. Next, subtype-specific marker genes and consensus clustering analyses also provided the transcriptional evidences for supporting the histological classifications by our signature. These results together suggested that our signature could rectify some misclassifications of NSCLC histological subtypes by current pathological assessment, especially for the clinical challenging cases, such as FFPE specimens with partial RNA degradation, mixed tumors with varied tumor cells proportions, small biopsy specimens with low-input mRNA specimens and poorly differentiated specimens, indicating the necessity for molecular classification.

Previously, several gene expression signatures to stratify ADC and SCC have been reported [1, 16, 36]. We additionally compared the performance of our signature with a previously reported 42-gene signature for stratifying ADC and SCC, which has more advantages than the other reported signatures [1]. The apparent accuracy of the 42-gene signature was 91.67% in the GSE19188 dataset with relative unambiguous NSCLC types, which was lower than that (94.44%) of our signature. In the other dataset (E-MTAB-2435) with relative unambiguous NSCLC types, the apparent accuracy of the two signatures was the same (98.41%). In the other test datasets, the total apparent accuracy of 42-gene signature was 91.87%, slightly lower than that (92.55%) of our signature. More importantly, the survival analyses showed that the OS difference between the SCC and ADC subtypes reclassified by our signature (log-rank *p* = 0.0012, HR = 1.60, 95% CI = 1.20–2.12, Fig. 3c) was more significant than that between the two histological subtypes reclassified by the 42-gene signature (log-rank *p* = 0.0130, HR = 1.45, 95% CI = 1.08–1.95, Additional file 1: Figure S8). The above results indicated that our signature could perform better than the 42-gene signature in rectifying some misclassifications by routine pathological assessment. The proliferation scores and gene expression patterns were not compared due to the small sample size of the inconsistent samples reclassified by the two signatures in each dataset. Moreover, comparing with the 42-gene signature, whose classification is dependent on the samples in its training dataset, our signature is much more convenient as it only needs detection of two genes within an individual and is independent of the other samples.

However, a limitation of this study is that most of the samples we used were diagnosed according to WHO 2004 criteria because few samples diagnosed according to WHO 2015 criteria could be found in public data. It is known that the current criteria auxiliary with immunostaining could reclassify a subset of cases diagnosed with the WHO 2004 criteria [37]. However, the choice of immunomarkers [37] and the subjective diagnosis of immunostaining results by pathologists have effects on the histological classification [38]. For the 23 poorly differentiated samples in the GSE94601 dataset, which were classified according to WHO 2015 criteria, our signature confirmed all the 19 pADC samples and reclassified two of the four pSCC samples as ADC. We provided tentatively evidences for supporting the reclassifications by our signature through estimating the proliferative activities of the two reclassified samples, based on the phenomenon that SCC has higher proliferative activity with poorer survival than ADC. In this study, we provided several indirect biological evidences to support the pathological reclassification of our signature, which should be further evaluated by collecting clinical samples for reevaluation by independent pathologists. Because HE staining is the conventional method for determining histological classification, the current qualitative signature, which is independent of the subjective diagnoses of pathologists, would be an auxiliary test for the ambiguous cases in the pathological assessment. Another limitation of this study is that the current signature can only distinguish ADC and SCC. We additionally applied the signature to the other subtypes of lung cancer (GSE60644), including three small cell lung cancer and nine large cell neuroendocrine carcinoma. The result showed that all these samples were reclassified into ADC group, indicating that the signature might have a better performance in distinguishing non-SCC from SCC, which should be instructive for treatment as most histology-dependent therapies for NSCLC are directed at ADC or non-squamous histological types [39]. It is noted that, the REO approach can handle more than 2 output classes [25], which merits exploration in the future.

## Conclusions

The non-subjective qualitative REOs signature would be an auxiliary test for the pathological assessment of the ambiguous cases, including the cases lacking the evidences of glandular or squamous differentiation. Notably, it is worthwhile to measure all possible qualitative transcriptional signatures together (“a panel for all”) for disease diagnosis, histological classification, prognosis and drug evaluation of lung cancer through a single whole (or a large panel of genes) RNA-sequencing for a sample to aid or even replace multiple conventional clinical detection, which could preserve precious tissue for the other molecular testing such as EGFR mutation detection.

## Methods

### The public data sources and data pre-processing

The 15 public gene expression datasets of NSCLC tissues (Table 1) were collected from Gene Expression Omnibus (GEO, http://www.ncbi.nlm.nih.gov/geo/), ArrayExpress (https://www.ebi.ac.uk/arrayexpress/) and The Cancer Genome Atlas (TCGA, http://cancergenome.nih.gov/).

The training data for extracting a REOs-based signature was integrated from two microarray datasets (GSE30219 and GSE18842), including 99 pathologically-determined ADC (denoted as pADC) samples, 92 pathologically-determined SCC (denoted as pSCC) samples and 59 normal controls.

In this study, 2 datasets (GSE19188 and E-MTAB-2435), were selected as “golden”standard datasets to test the performance of the signature, as their histological classifications were concordantly determined by two independent pathologists, whose subtypes were relative unambiguous. Seven datasets recording survival information were integrated for survival analyses, including 805 pADC and 125 pSCC samples of stage I-III patients treated with curative surgery resection only. The clinical information of the 7 datasets is displayed in Additional file 1: Table S1. The signature was also tested in FFPE specimens with partial RNA degradation (GSE44170), mixed tumors with varied proportions of tumor cells (TCGA-ADC, TCGA-SCC), small biopsy specimens with low-input RNA (GSE58661) and poorly differentiated specimens (GSE94601). Notably, the GSE94601 dataset includes 19 poorly differentiated pADC and 4 poorly differentiated pSCC samples, which were improperly assigned to LCC subtype before and reclassified by the authors using ADC and SCC immunomarkers according to the WHO 2015 criteria [40]. Except these reclassified LCC samples, histological classifications of the other tumors in the above datasets were diagnosed according to the WHO 2004 criteria [1].

For data generated by the Affymetrix microarray platforms, the Robust Multi-array Average algorithm [41] was used for preprocessing the raw data and the other datasets generated by the Illumina platforms, the originally processed data (series matrix files) were used. Probe IDs were mapped to Gene IDs using the corresponding platform files. For each sample, the expression measurements of all probes corresponding to the same Gene ID were averaged to obtain a single measurement. Probes that did not match any Gene ID or matched multiple Gene IDs were deleted. For TCGA data (ADC/SCC), the normalized count values of level 3 gene expression data derived from Illumina HiSeqV2 were extracted as gene expression measurements.

### Developing the signature for distinguishing ADC from SCC

First, Significance Analysis of Microarrays (SAM) algorithm [42] was used to identify differentially expressed genes (DE genes) in pADC and pSCC respectively, when compared with normal controls. The *p* values were adjusted using the Benjamini-Hochberg procedure for multiple testing to control the false discovery rate (FDR) [43]. We selected the genes which were differentially expressed in both pADC and pSCC but with opposite dysregulated directions when compared with normal controls, and defined them as subtype-opposite genes.

Then, for a pair of genes (*a* and *b*) both derived from the subtype-opposite genes, Fisher’s exact test was used to evaluate whether the frequency of pSCC samples with a specific REO pattern (E*a* > E*b*), where E*a* and E*b* represent the expression levels of gene *a* and *b*, respectively, was significantly higher than the frequency in pADC samples. The significant gene pairs were defined as subtype-opposite gene pairs.

Finally, for each subtype-opposite gene pair, we calculated the apparent accuracy (Formula 1) of the gene pair for distinguishing ADC from SCC, as the pathological assessments are not 100% reliable [29]. We chose each of the top 50 gene pairs ranked according to the apparent accuracy as a seed and performed forward selection procedure to iteratively added one subtype-opposite gene pair that achieved the highest apparent accuracy, based on the classification rule as follows: a sample was determined to be SCC if more than half of the REOs of the gene pairs within this sample voted for SCC, otherwise ADC. Among the results derived from all of the seeds, a set of gene pairs with the highest apparent accuracy was chosen as the signature for distinguishing ADC from SCC. If several sets of gene pairs achieved the highest apparent accuracy, then the set with the fewest gene pairs (primary condition) and the largest median absolute rank difference of gene pairs in pSCC and pADC samples (secondary condition) was selected as signature. Here, the absolute rank difference for each gene pair (G*a* and G*b*) between the two groups was calculated as Formula 2.

1 $$ \mathrm{apparent}\ \mathrm{accuracy}=\mathrm{C}/\mathrm{M}\ast 100\% $$

where C is the number of samples which were accurately classified by the signature when compared with their original histological classification, and M is the total number of samples used in the dataset. Here, M is 191 which is the total number of the pADC and pSCC samples in the training dataset.

2 $$ {\overline{R}}_{ab}=\sqrt{{\overline{R}}_{ab(pADC)}{\overline{R}}_{ab(pSCC)}} $$

where ${\overline{R}}_{ab(pADC)}$ and ${\overline{R}}_{ab(pSCC)}$ are the geometric means of the absolute rank differences of the gene pair (G*a* and G*b*) in all samples of pADC and pSCC groups, respectively.

### Immunohistochemical analysis of lung cancer tissue microarrays

The lung cancer array for immunohistochemical analysis was bought from Anenabio, Xi’an, China (Cat#LC2161), including 96 pADC samples and 80 pSCC samples. The slides of human lung cancer tissue microarray used in this study were dewaxed with xylene and rehydrated through gradient ethanol into water. Antigens were unmasked by 10 mM citric acid buffer. Endogenous peroxidase activity was blocked by 3% hydrogen peroxidase. After further blocking for non-specific binding, the slides were incubated with specific anti-*Krt5* (#AF0194, Affinity) or anti-*Agr2* (ab76473, Abcam) primary antibody and then with secondary antibody conjugated with HRP. The slides were developed with DAB substrates, counterstained with haematoxylin, dehydrated and mounted with neutral balsam. Each tissue sample of the microarray was then imaged under miscroscope Carl Zeiss GmbH with same settings. The protein expression was quantified and scored basing on a multiplicative index of the average staining intensity (1–4) and the extent of staining (1–4 in the cores, yielding a 16-point staining index that ranged from 1 (no staining) to 16 (extensive, strong staining). The staining intensity was scored as follows: 1, negative; 2, weak; 3, moderate; and 4, strong. The extent of staining was scored as follows: 1, less than 10% positive cells; 2, 10 to 30%; 3, 30 to 70%; and 4, more than 70%. Score of staining index less than 5 was considered low expression, score of 5–10 considered medium expression and score of 11–16 considered high expression. Fisher’s exact test was used to compare the frequencies of the *Krt5* and *Agr2* proteins expressions between 96 pADC samples and 80 pSCC samples.

### Survival analyses

To avoid the bias of patient follow-up duration among different datasets, the overall survival (OS) of patients were truncated at 60 months. Survival curves were estimated using the Kaplan-Meier method and were compared using the log-rank test [44]. A multivariate Cox proportional-hazards regression model [45] was used to assess whether the reclassified groups was independently associated with patient survival after adjusting for data centers and clinical parameters, including stage, age and gender. Hazard ratios (HRs) and 95% confidence intervals (CIs) were generated using univariate and multivariate Cox proportional hazards model.

### Proliferation score calculation

The proliferation score for each sample was calculated by averaging the expressions of the 44 genes in a proliferation signature [31] in a cohort. A higher proliferation score indicates more proliferative activity. Wilcoxon rank sum test was used to test the difference of proliferation scores between two groups.

### Differential expression and consensus clustering analyses

The RankProd (RP) algorithm [46], which is a non-parametric test, was used to estimate whether the subtype-specific marker genes were differentially expressed between reclassified samples and signature-confirmed samples. The subtype-specific marker genes include SCC marker genes (*KRT5*, *TP63*) [11], ADC marker genes (*NAPSA*, *TTF1*) [11] and neuroendocrine marker genes (*CD56*, *SYP*, *CHGA*) [47].

Consensus clustering was performed using the ConsensusClusterPlus package according to the Ward method for hierarchical clustering [48]. The samples were clustered using top 1000 most variable genes across all the pADC and pSCC samples in a cohort, which were determined by the median absolute deviation. Briefly, the expression data for each of the top 1000 genes were first transformed to Z-scores, and then subsampled 80% of samples (items) and genes (features) 2000 times and partitioned each subsample up into *k* = 10 groups (*k* represents the number of clusters) by the agglomerative hierarchical clustering algorithm using Pearson correlation distance. To identify the optimum number of clusters, *k* corresponding to the first downwards inflection in cumulative distribution function (CDF) was used. Additionally, we also performed the consensus clustering using top 2000 and 3000 most variable genes, respectively.

All statistical analyses were performed using the R 2.15.3 (http://www.r-project.org/).

## Supplementary information

**Additional file 1: Table S1.** Clinical characteristics of patients treated with curative surgery resection only. **Table S2.** The top 50 subtype-opposite gene pairs. **Table S3.** The histological classification of samples classified by pathological assessment, signature and clustering in the GSE50081. **Table S4.** The histological classification of samples classified by pathological assessment, signature and cluster in the GSE58661. **Figure S1.** Kaplan-Meier curves of OS respectively for the SCC and ADC groups reclassified by the signature and original pathological assessment in the data integrated by 6 datasets. **Figure S2.** The consensus clustering of the samples based on top 1000 most variable genes in the GSE50081 dataset. **Figure S3.** The consensus clustering of the samples based on top 2000 most variable genes in the GSE50081 dataset. **Figure S4.** The consensus clustering of the samples based on top 3000 most variable genes in the GSE50081 dataset. **Figure S5.** The consensus clustering of the samples based on top 1000 most variable genes in the GSE58661 dataset for small biopsy specimens. **Figure S6.** The consensus clustering of the samples based on top 2000 most variable genes in the GSE58661 dataset for small biopsy specimens. **Figure S7.** The consensus clustering of the samples based on top 3000 most variable genes in the GSE58661 dataset for small biopsy specimens. **Figure S8.** The Kaplan-Meier curves of overall survival respectively for the ADC and SCC groups of patients treated with curative surgery resection only.

## Data Availability

The datasets analyzed during the current study are available in the Gene Expression Omnibus (GEO, http://www.ncbi.nlm.nih.gov/geo/), ArrayExpress (https://www.ebi.ac.uk/arrayexpress/) and The Cancer Genome Atlas (TCGA, http://cancergenome.nih.gov/).

## Abbreviations

ADC: Adenocarcinoma
CIs: Confidence intervals
DE genes: Differentially expressed genes
EGFR: Epidermal growth factor receptor
FDR: False discovery rate
FFPE: Formalin fixed paraffin-embedded
GEO: Gene Expression Omnibus
HRs: Hazard ratios
LCC: Large cell carcinoma
NSCLC: Non-small cell lung cancer
NSCLC-NOS: NSCLC-not otherwise specified
OS: Overall survival
pADC: Pathologically-determined ADC
pSCC: Pathologically-determined SCC
REOs: Relative expression orderings
RP: RankProd
SAM: Significance Analysis of Microarrays
SCC: Squamous cell carcinoma
TCGA: The Cancer Genome Atlas
