# Supplementary material for: A qualitative transcriptional signature for the histological reclassification of lung squamous cell carcinomas and adenocarcinomas
Source: BMC Genomics. 2019 Nov 21;20:881. doi: 10.1186/s12864-019-6086-2 (PMC6868745; doi:10.1186/s12864-019-6086-2)
Supplement: Supplementary file 1 — Additional file 1: Table S1. Clinical characteristics of patients treated with curative surgery resection only. Table S2. The top 50 subtype-opposite gene pairs. Table S3. The histological classification of samples classified by pathological assessment, signature and clustering in the GSE50081. Table S4. The histological classification of samples classified by pathological assessment, signature and cluster in the GSE58661. Figure S1. Kaplan-Meier curves of OS respectively for the SCC and ADC groups reclassified by the signature and original pathological assessment in the data integrated by 6 datasets. Figure S2. The consensus clustering of the samples based on top 1000 most variable genes in the GSE50081 dataset. Figure S3. The consensus clustering of the samples based on top 2000 most variable genes in the GSE50081 dataset. Figure S4. The consensus clustering of the samples based on top 3000 most variable genes in the GSE50081 dataset. Figure S5. The consensus clustering of the samples based on top 1000 most variable genes in the GSE58661 dataset for small biopsy specimens. Figure S6. The consensus clustering of the samples based on top 2000 most variable genes in the GSE58661 dataset for small biopsy specimens. Figure S7. The consensus clustering of the samples based on top 3000 most variable genes in the GSE58661 dataset for small biopsy specimens. Figure S8. The Kaplan-Meier curves of overall survival respectively for the ADC and SCC groups of patients treated with curative surgery resection only. [file 12864_2019_6086_MOESM1_ESM.docx]

**Additional file 1: Table S1. Clinical characteristics of patients treated with curative surgery resection only**

| **Data Source** | **Year** | **Subtype** | **Sample** | **Age** | | **Gender** | | **Stage** | | |
| --- | --- | --- | --- | --- | --- | --- | --- | --- | --- | --- |
|  |  |  |  | Age ≤ 65 | Age > 65 | Female | Male | I | II | III |
| **GSE42127** | 2013 | pSCC | 32 | 11 | 21 | 14 | 18 | 18 | 7 | 7 |
|  |  | pADC | 90 | 40 | 50 | 48 | 42 | 65 | 16 | 9 |
| **GSE50081** | 2013 | pSCC | 43 | 15 | 28 | 18 | 25 | 27 | 16 | 0 |
|  |  | pADC | 127 | 40 | 87 | 62 | 65 | 92 | 35 | 0 |
| **GSE37745** | 2012 | pSCC | 24 | 8 | 16 | 8 | 16 | 15 | 4 | 5 |
|  |  | pADC | 40 | 16 | 24 | 25 | 15 | 31 | 4 | 5 |
| **GSE31210** | 2011 | pSCC | 0 | 0 | 0 | 0 | 0 | 0 | 0 | 0 |
|  |  | pADC | 204 | 157 | 47 | 109 | 95 | 162 | 42 | 0 |
| **GSE31546** | 2011 | pSCC | 0 | 0 | 0 | 0 | 0 | 0 | 0 | 0 |
|  |  | pADC | 13 | 13 | 0 | 10 | 3 | 13 | 0 | 0 |
| **GSE14814** | 2010 | pSCC | 26 | 16 | 10 | 3 | 23 | 12 | 14 | 0 |
|  |  | pADC | 32 | 24 | 8 | 14 | 18 | 20 | 12 | 0 |
| **GSE68465** | 2008 | pSCC | 0 | 0 | 0 | 0 | 0 | 0 | 0 | 0 |
|  |  | pADC | 299 | 145 | 154 | 145 | 154 | 211 | 51 | 37 |

**Table S2. The top 50 subtype-opposite gene pairs**

| **Seed**  **Gene *a*, Gene *b*** | **Seed in Training**^#^ | **Set** | **Set in Training**^#^ | **Set in GSE19188**^#^ | **Set in**  **E-MTAB-2435**^#^ |
| --- | --- | --- | --- | --- | --- |
| [*KRT5*, *AGR2*] | 98.43% | [*KRT5*, *AGR2*] | 98.43% | 94.44% | 98.41% |
| [*KRT5*, *CLDN7*] | 97.91% | [*KRT5*, *CLDN7*] | 97.91% | 91.67% | 95.24% |
| [*KRT5*, *CTBP2*] | 97.91% | [*KRT5*, *CTBP2*] | 97.91% | 93.06% | 93.65% |
| [*KRT5*, *IRF7*] | 97.91% | [*KRT5*, *IRF7*] | 97.91% | 91.67% | 98.41% |
| [*KRT5*, *RASSF7*] | 97.91% | [*KRT5*, *RASSF7*] | 97.91% | 90.28% | 96.83% |
| [*KRT5*, *CDK2AP2*] | 97.91% | [*KRT5*, *CDK2AP2*] | 97.91% | 91.67% | 96.83% |
| [*KRT5*, *ATG2A*] | 97.91% | [*KRT5*, *ATG2A*] | 97.91% | 90.28% | 96.83% |
| [*KRT5*, *CXXC5*] | 97.91% | [*KRT5*, *CXXC5*] | 97.91% | 91.67% | 96.83% |
| [*KRT5*, *GOLPH3L*] | 97.91% | [*KRT5*, *GOLPH3L*] | 97.91% | 93.06% | 95.24% |
| [*CLCA2*, *ADRA2A*] | 97.38% | [*CLCA2*, *ADRA2A*] | 98.43% | 91.67% | 98.41% |
| [*KRT5*, *EFNA1*] | 97.38% | [*KRT5*, *EFNA1*] | 97.38% | 94.44% | 93.65% |
| [*STON2*, *GPR39*] | 97.38% | [*STON2*, *GPR39*] | 97.38% | 86.11% | 98.41% |
| [*KRT5*, *IFNAR2*] | 97.38% | [*KRT5*, *IFNAR2*] | 97.38% | 91.67% | 98.41% |
| [*KRT5*, *P2RX4*] | 97.38% | [*KRT5*, *P2RX4*] | 97.38% | 93.06% | 96.83% |
| [*KRT5*, *PAM*] | 97.38% | [*KRT5*, *PAM*] | 97.38% | 93.06% | 93.65% |
| [*KRT5*, *PLS1*] | 97.38% | [*KRT5*, *PLS1*] | 97.38% | 93.06% | 93.65% |
| [*KRT5*, *PRNP*] | 97.38% | [*KRT5*, *PRNP*] | 97.38% | 93.06% | 95.24% |
| [*KRT5*, *PRSS8*] | 97.38% | [*KRT5*, *PRSS8*;  *KRT5*, *AGR2*] | 98.43% | 94.44% | 96.83% |
| [*KRT5*, *RALB*] | 97.38% | [*KRT5*, *RALB*] | 97.38% | 91.67% | 93.65% |
| [*KRT5*, *SOAT1*] | 97.38% | [*KRT5*, *SOAT1*] | 97.38% | 93.06% | 95.24% |
| [*KRT5*, *OGT*] | 97.38% | [*KRT5*, *OGT*] | 97.38% | 90.28% | 96.83% |
| [*KRT5*, *FAM50A*] | 97.38% | [*KRT5*, *FAM50A*] | 97.38% | 93.06% | 93.65% |
| [*KRT5*, *LZTS3*] | 97.38% | [*KRT5*, *LZTS3*] | 97.38% | 91.67% | 96.83% |
| [*KRT5*, *SLC30A9*] | 97.38% | [*KRT5*, *SLC30A9*] | 97.38% | 93.06% | 96.83% |
| [*KRT5*, *CYB561D2*] | 97.38% | [*KRT5*, *CYB561D2*] | 97.38% | 90.28% | 98.41% |
| [*KRT5*, *MGRN1*] | 97.38% | [*KRT5*, *MGRN1*] | 97.38% | 91.67% | 98.41% |
| [*KRT5*, *TRAM1*] | 97.38% | [*KRT5*, *TRAM1*] | 97.38% | 94.44% | 84.13% |
| [*KRT5*, *HEATR5A*] | 97.38% | [*KRT5*, *HEATR5A*] | 97.38% | 91.67% | 98.41% |
| [*KRT5*, *SLC43A3*] | 97.38% | [*KRT5*, *SLC43A3*] | 97.38% | 93.06% | 98.41% |
| [*KRT5*, *INSIG2*] | 97.38% | [*KRT5*, *INSIG2*] | 97.38% | 93.06% | 95.24% |
| [*KRT5*, *CKLF*] | 97.38% | [*KRT5*, *CKLF*] | 97.38% | 93.06% | 96.83% |
| [*KRT5*, *ERRFI1*] | 97.38% | [*KRT5*, *ERRFI1*] | 97.38% | 94.44% | 93.65% |
| [*KRT5*, *RFK*] | 97.38% | [*KRT5*, *RFK*] | 97.38% | 93.06% | 93.65% |
| [*KRT5*, *TMEM176A*] | 97.38% | [*KRT5*, *TMEM176A*] | 97.38% | 93.06% | 96.83% |
| [*KRT5*, *SIL1*] | 97.38% | [*KRT5*, *SIL1*] | 97.38% | 91.67% | 96.83% |
| [*KRT5*, *EMB*] | 97.38% | [*KRT5*, *EMB*] | 97.38% | 93.06% | -- |
| [*KRT5*, *UBXN2A*] | 97.38% | [*KRT5*, *UBXN2A*] | 97.38% | 91.67% | 93.65% |
| [*KRT5*, *LINC00623*] | 97.38% | [*KRT5*, *LINC00623*] | 97.38% | 91.67% | -- |
| [*STON2*, *SNTB1*] | 97.38% | [*STON2*, *SNTB1*] | 97.38% | 86.11% | 92.06% |
| [*CLCA2*, *INPP4B*] | 97.38% | [*CLCA2*, *INPP4B*] | 97.38% | 91.67% | 98.41% |
| [*CLCA2*, *PNMA2*] | 97.38% | [*CLCA2*, *PNMA2*] | 97.38% | 94.44% | 93.65% |
| [*KRT5*, *ATP6V1E1*] | 96.86% | [*KRT5*, *ATP6V1E1*] | 96.86% | 93.06% | 93.65% |
| [*CLCA2*, *AZGP1*] | 96.86% | [*CLCA2*, *AZGP1*] | 96.86% | 93.06% | 93.65% |
| [*KRT5*, *DUT*] | 96.86% | [*KRT5*, *DUT*] | 96.86% | 93.06% | 93.65% |
| [*CLCA2*, *GLRB*] | 96.86% | [*CLCA2*, *GLRB*] | 96.86% | 87.50% | 95.24% |
| [*CLCA2*, *CCR10*] | 96.86% | [*CLCA2*, *CCR10*;  [*KRT5*, *AGR2*] | 97.91% | 94.44% | 93.65% |
| [*KRT5*, *MGST1*] | 96.86% | [*KRT5*, *MGST1*] | 96.86% | 94.44% | 93.65% |
| [*KRT5*, *NDUFA6*] | 96.86% | [*KRT5*, *NDUFA6*] | 96.86% | 93.06% | 93.65% |
| [*KRT5*, *PON2*] | 96.86% | [*KRT5*, *PON2*;  *KRT5*, *AGR2*] | 97.91% | 94.44% | 96.83% |
| [*KRT5*, *PSMA2*] | 96.86% | [*KRT5*, *PSMA2*] | 96.86% | 94.44% | 93.65% |

^#^ represents the apparent accuracy of each seed gene pair or set.

**Table S3. The histological classification of samples classified by pathological assessment, signature and clustering in the GSE50081**

| **No.** | **Sampleid** | **Pathological lable** | **Signature label** | **TOP 1000 cluster label** | **TOP 2000 cluster label** | **TOP 3000 cluster label** |
| --- | --- | --- | --- | --- | --- | --- |
| 1 | GSM1213670 | ADC | ADC | ADC-like | ADC-like | ADC-like |
| 2 | GSM1213671 | ADC | ADC | ADC-like | ADC-like | ADC-like |
| 3 | GSM1213672 | ADC | ADC | ADC-like | ADC-like | ADC-like |
| 4 | GSM1213673 | ADC | ADC | SCC-like | ADC-like | ADC-like |
| 5 | GSM1213674 | SCC | SCC | SCC-like | ADC-like | ADC-like |
| 6 | GSM1213675 | SCC | SCC | SCC-like | SCC-like | SCC-like |
| 7 | GSM1213676 | ADC | ADC | ADC-like | ADC-like | ADC-like |
| **8** | **GSM1213677** | **ADC** | **SCC** | **SCC-like** | **SCC-like** | **SCC-like** |
| 9 | GSM1213678 | ADC | ADC | ADC-like | ADC-like | ADC-like |
| 10 | GSM1213679 | ADC | ADC | ADC-like | ADC-like | ADC-like |
| 11 | GSM1213680 | ADC | ADC | ADC-like | ADC-like | ADC-like |
| 12 | GSM1213681 | ADC | ADC | ADC-like | ADC-like | ADC-like |
| 13 | GSM1213682 | ADC | ADC | ADC-like | ADC-like | ADC-like |
| 14 | GSM1213683 | ADC | ADC | ADC-like | ADC-like | ADC-like |
| 15 | GSM1213684 | SCC | SCC | SCC-like | SCC-like | ADC-like |
| 16 | GSM1213685 | ADC | ADC | ADC-like | ADC-like | ADC-like |
| 17 | GSM1213686 | ADC | ADC | ADC-like | ADC-like | ADC-like |
| 18 | GSM1213687 | ADC | ADC | ADC-like | ADC-like | ADC-like |
| 19 | GSM1213688 | ADC | ADC | ADC-like | ADC-like | ADC-like |
| 20 | GSM1213689 | ADC | ADC | ADC-like | ADC-like | ADC-like |
| 21 | GSM1213690 | ADC | ADC | ADC-like | ADC-like | ADC-like |
| 22 | GSM1213691 | ADC | ADC | ADC-like | ADC-like | ADC-like |
| 23 | GSM1213692 | ADC | ADC | ADC-like | ADC-like | ADC-like |
| 24 | GSM1213693 | ADC | ADC | ADC-like | ADC-like | ADC-like |
| 25 | GSM1213694 | ADC | ADC | ADC-like | ADC-like | ADC-like |
| 26 | GSM1213695 | ADC | ADC | SCC-like | ADC-like | ADC-like |
| 27 | GSM1213696 | ADC | ADC | ADC-like | ADC-like | ADC-like |
| 28 | GSM1213697 | ADC | ADC | ADC-like | ADC-like | ADC-like |
| 29 | GSM1213698 | ADC | ADC | SCC-like | ADC-like | ADC-like |
| 30 | GSM1213699 | ADC | ADC | ADC-like | ADC-like | ADC-like |
| 31 | GSM1213700 | ADC | ADC | ADC-like | ADC-like | ADC-like |
| 32 | GSM1213701 | SCC | SCC | SCC-like | SCC-like | SCC-like |
| 33 | GSM1213702 | ADC | ADC | ADC-like | ADC-like | ADC-like |
| 34 | GSM1213703 | ADC | ADC | ADC-like | ADC-like | ADC-like |
| 35 | GSM1213704 | ADC | ADC | ADC-like | ADC-like | ADC-like |
| 36 | GSM1213705 | SCC | SCC | SCC-like | SCC-like | SCC-like |
| 37 | GSM1213706 | SCC | SCC | SCC-like | SCC-like | SCC-like |
| 38 | GSM1213707 | ADC | ADC | ADC-like | ADC-like | ADC-like |
| 39 | GSM1213708 | SCC | SCC | SCC-like | SCC-like | SCC-like |
| 40 | GSM1213709 | SCC | SCC | SCC-like | SCC-like | SCC-like |
| 41 | GSM1213710 | ADC | ADC | SCC-like | ADC-like | ADC-like |
| 42 | GSM1213711 | SCC | SCC | SCC-like | SCC-like | SCC-like |
| 43 | GSM1213712 | SCC | SCC | SCC-like | SCC-like | SCC-like |
| 44 | GSM1213714 | SCC | SCC | SCC-like | SCC-like | SCC-like |
| **45** | **GSM1213715** | **ADC** | **SCC** | **SCC-like** | **SCC-like** | **SCC-like** |
| **46** | **GSM1213716** | **ADC** | **SCC** | **SCC-like** | **SCC-like** | **SCC-like** |
| 47 | GSM1213717 | ADC | ADC | ADC-like | ADC-like | SCC-like |
| 48 | GSM1213718 | SCC | SCC | SCC-like | SCC-like | SCC-like |
| **49** | **GSM1213719** | **SCC** | **ADC** | **ADC-like** | **ADC-like** | **ADC-like** |
| 50 | GSM1213720 | ADC | ADC | ADC-like | ADC-like | ADC-like |
| **51** | **GSM1213722** | **ADC** | **SCC** | **SCC-like** | **SCC-like** | **SCC-like** |
| 52 | GSM1213723 | ADC | ADC | ADC-like | ADC-like | SCC-like |
| 53 | GSM1213724 | ADC | ADC | ADC-like | ADC-like | ADC-like |
| 54 | GSM1213726 | ADC | ADC | ADC-like | ADC-like | ADC-like |
| 55 | GSM1213727 | ADC | ADC | SCC-like | ADC-like | ADC-like |
| 56 | GSM1213728 | ADC | ADC | ADC-like | ADC-like | ADC-like |
| 57 | GSM1213729 | SCC | SCC | SCC-like | SCC-like | SCC-like |
| 58 | GSM1213730 | ADC | ADC | ADC-like | ADC-like | SCC-like |
| 59 | GSM1213731 | SCC | SCC | SCC-like | SCC-like | SCC-like |
| 60 | GSM1213732 | SCC | SCC | SCC-like | SCC-like | SCC-like |
| 61 | GSM1213733 | ADC | ADC | ADC-like | ADC-like | SCC-like |
| 62 | GSM1213734 | ADC | ADC | ADC-like | ADC-like | ADC-like |
| 63 | GSM1213735 | ADC | ADC | ADC-like | ADC-like | ADC-like |
| 64 | GSM1213736 | ADC | ADC | SCC-like | ADC-like | ADC-like |
| 65 | GSM1213737 | ADC | ADC | ADC-like | ADC-like | ADC-like |
| 66 | GSM1213738 | ADC | ADC | ADC-like | ADC-like | ADC-like |
| 67 | GSM1213739 | ADC | ADC | ADC-like | ADC-like | ADC-like |
| 68 | GSM1213740 | ADC | ADC | ADC-like | ADC-like | ADC-like |
| 69 | GSM1213741 | ADC | ADC | ADC-like | ADC-like | ADC-like |
| 70 | GSM1213742 | ADC | ADC | ADC-like | ADC-like | ADC-like |
| 71 | GSM1213743 | ADC | ADC | ADC-like | ADC-like | ADC-like |
| 72 | GSM1213744 | SCC | SCC | SCC-like | SCC-like | SCC-like |
| 73 | GSM1213746 | ADC | ADC | ADC-like | ADC-like | ADC-like |
| 74 | GSM1213747 | ADC | ADC | ADC-like | ADC-like | ADC-like |
| 75 | GSM1213748 | SCC | SCC | SCC-like | SCC-like | SCC-like |
| 76 | GSM1213749 | ADC | ADC | ADC-like | ADC-like | ADC-like |
| **77** | **GSM1213750** | **ADC** | **SCC** | **ADC-like** | **ADC-like** | **SCC-like** |
| 78 | GSM1213751 | ADC | ADC | ADC-like | ADC-like | ADC-like |
| 79 | GSM1213752 | ADC | ADC | ADC-like | ADC-like | ADC-like |
| 80 | GSM1213753 | ADC | ADC | ADC-like | ADC-like | ADC-like |
| 81 | GSM1213754 | ADC | ADC | SCC-like | ADC-like | ADC-like |
| 82 | GSM1213756 | ADC | ADC | ADC-like | ADC-like | ADC-like |
| 83 | GSM1213757 | ADC | ADC | ADC-like | ADC-like | ADC-like |
| **84** | **GSM1213758** | **ADC** | **SCC** | **ADC-like** | **ADC-like** | **ADC-like** |
| **85** | **GSM1213759** | **ADC** | **SCC** | **ADC-like** | **ADC-like** | **ADC-like** |
| 86 | GSM1213760 | ADC | ADC | ADC-like | ADC-like | SCC-like |
| 87 | GSM1213761 | ADC | ADC | ADC-like | ADC-like | ADC-like |
| 88 | GSM1213762 | ADC | ADC | ADC-like | ADC-like | SCC-like |
| 89 | GSM1213763 | ADC | ADC | SCC-like | ADC-like | ADC-like |
| 90 | GSM1213764 | ADC | ADC | ADC-like | ADC-like | SCC-like |
| 91 | GSM1213765 | SCC | SCC | ADC-like | ADC-like | ADC-like |
| 92 | GSM1213766 | SCC | SCC | SCC-like | SCC-like | SCC-like |
| 93 | GSM1213767 | SCC | SCC | SCC-like | SCC-like | SCC-like |
| 94 | GSM1213768 | ADC | ADC | ADC-like | ADC-like | SCC-like |
| 95 | GSM1213769 | ADC | ADC | ADC-like | ADC-like | ADC-like |
| 96 | GSM1213770 | ADC | ADC | ADC-like | ADC-like | ADC-like |
| 97 | GSM1213771 | SCC | SCC | SCC-like | SCC-like | SCC-like |
| 98 | GSM1213772 | SCC | SCC | SCC-like | ADC-like | ADC-like |
| 99 | GSM1213773 | ADC | ADC | ADC-like | ADC-like | ADC-like |
| **100** | **GSM1213774** | **ADC** | **SCC** | **SCC-like** | **ADC-like** | **ADC-like** |
| 101 | GSM1213775 | ADC | ADC | ADC-like | ADC-like | ADC-like |
| 102 | GSM1213777 | ADC | ADC | ADC-like | ADC-like | ADC-like |
| 103 | GSM1213778 | SCC | SCC | SCC-like | SCC-like | SCC-like |
| 104 | GSM1213779 | ADC | ADC | ADC-like | ADC-like | ADC-like |
| 105 | GSM1213780 | ADC | ADC | ADC-like | ADC-like | ADC-like |
| **106** | **GSM1213781** | **ADC** | **SCC** | **ADC-like** | **ADC-like** | **ADC-like** |
| 107 | GSM1213782 | ADC | ADC | ADC-like | ADC-like | ADC-like |
| 108 | GSM1213783 | ADC | ADC | ADC-like | ADC-like | SCC-like |
| 109 | GSM1213784 | ADC | ADC | ADC-like | ADC-like | ADC-like |
| 110 | GSM1213785 | ADC | ADC | ADC-like | ADC-like | ADC-like |
| 111 | GSM1213786 | SCC | SCC | SCC-like | SCC-like | SCC-like |
| 112 | GSM1213787 | ADC | ADC | ADC-like | ADC-like | ADC-like |
| 113 | GSM1213788 | ADC | ADC | ADC-like | ADC-like | ADC-like |
| 114 | GSM1213789 | SCC | SCC | SCC-like | SCC-like | SCC-like |
| 115 | GSM1213790 | SCC | SCC | SCC-like | ADC-like | SCC-like |
| 116 | GSM1213791 | SCC | SCC | SCC-like | SCC-like | SCC-like |
| 117 | GSM1213792 | ADC | ADC | ADC-like | ADC-like | ADC-like |
| 118 | GSM1213793 | ADC | ADC | SCC-like | ADC-like | ADC-like |
| 119 | GSM1213794 | ADC | ADC | ADC-like | ADC-like | ADC-like |
| 120 | GSM1213795 | ADC | ADC | ADC-like | ADC-like | ADC-like |
| 121 | GSM1213796 | SCC | SCC | SCC-like | SCC-like | SCC-like |
| 122 | GSM1213797 | ADC | ADC | ADC-like | ADC-like | ADC-like |
| 123 | GSM1213798 | ADC | ADC | ADC-like | ADC-like | ADC-like |
| 124 | GSM1213800 | ADC | ADC | ADC-like | ADC-like | ADC-like |
| **125** | **GSM1213801** | **ADC** | **SCC** | **SCC-like** | **ADC-like** | **ADC-like** |
| 126 | GSM1213802 | SCC | SCC | SCC-like | SCC-like | SCC-like |
| 127 | GSM1213803 | ADC | ADC | ADC-like | ADC-like | ADC-like |
| 128 | GSM1213804 | ADC | ADC | ADC-like | ADC-like | ADC-like |
| 129 | GSM1213805 | ADC | ADC | SCC-like | ADC-like | ADC-like |
| 130 | GSM1213806 | ADC | ADC | SCC-like | ADC-like | ADC-like |
| 131 | GSM1213807 | ADC | ADC | ADC-like | SCC-like | SCC-like |
| 132 | GSM1213808 | ADC | ADC | ADC-like | ADC-like | ADC-like |
| 133 | GSM1213809 | ADC | ADC | ADC-like | ADC-like | ADC-like |
| 134 | GSM1213810 | ADC | ADC | ADC-like | ADC-like | ADC-like |
| 135 | GSM1213811 | ADC | ADC | ADC-like | ADC-like | ADC-like |
| **136** | **GSM1213812** | **ADC** | **SCC** | **SCC-like** | **SCC-like** | **SCC-like** |
| 137 | GSM1213813 | SCC | SCC | SCC-like | SCC-like | SCC-like |
| 138 | GSM1213814 | ADC | ADC | ADC-like | ADC-like | ADC-like |
| 139 | GSM1213815 | SCC | SCC | SCC-like | SCC-like | SCC-like |
| 140 | GSM1213816 | ADC | ADC | ADC-like | ADC-like | SCC-like |
| **141** | **GSM1213817** | **SCC** | **ADC** | **ADC-like** | **ADC-like** | **ADC-like** |
| 142 | GSM1213818 | ADC | ADC | ADC-like | ADC-like | ADC-like |
| **143** | **GSM1213819** | **ADC** | **SCC** | **SCC-like** | **SCC-like** | **SCC-like** |
| 144 | GSM1213820 | ADC | ADC | ADC-like | ADC-like | ADC-like |
| **145** | **GSM1213821** | **SCC** | **ADC** | **SCC-like** | **ADC-like** | **ADC-like** |
| 146 | GSM1213822 | SCC | SCC | SCC-like | SCC-like | SCC-like |
| 147 | GSM1213824 | ADC | ADC | ADC-like | ADC-like | ADC-like |
| 148 | GSM1213825 | ADC | ADC | ADC-like | ADC-like | ADC-like |
| 149 | GSM1213826 | ADC | ADC | SCC-like | ADC-like | ADC-like |
| 150 | GSM1213827 | ADC | ADC | ADC-like | ADC-like | ADC-like |
| **151** | **GSM1213828** | **SCC** | **ADC** | **ADC-like** | **ADC-like** | **ADC-like** |
| 152 | GSM1213829 | SCC | SCC | SCC-like | SCC-like | SCC-like |
| 153 | GSM1213830 | SCC | SCC | SCC-like | SCC-like | SCC-like |
| 154 | GSM1213831 | ADC | ADC | ADC-like | ADC-like | ADC-like |
| 155 | GSM1213833 | ADC | ADC | ADC-like | ADC-like | ADC-like |
| 156 | GSM1213834 | ADC | ADC | SCC-like | ADC-like | ADC-like |
| **157** | **GSM1213835** | **SCC** | **ADC** | **ADC-like** | **SCC-like** | **SCC-like** |
| 158 | GSM1213836 | ADC | ADC | ADC-like | ADC-like | ADC-like |
| **159** | **GSM1213837** | **ADC** | **SCC** | **SCC-like** | **SCC-like** | **SCC-like** |
| **160** | **GSM1213838** | **SCC** | **ADC** | **ADC-like** | **ADC-like** | **ADC-like** |
| 161 | GSM1213839 | SCC | SCC | SCC-like | SCC-like | SCC-like |
| 162 | GSM1213840 | ADC | ADC | ADC-like | ADC-like | ADC-like |
| 163 | GSM1213841 | SCC | SCC | SCC-like | SCC-like | SCC-like |
| 164 | GSM1213843 | SCC | SCC | SCC-like | SCC-like | SCC-like |
| 165 | GSM1213844 | ADC | ADC | ADC-like | ADC-like | ADC-like |
| **166** | **GSM1213845** | **ADC** | **SCC** | **ADC-like** | **ADC-like** | **SCC-like** |
| 167 | GSM1213846 | ADC | ADC | SCC-like | ADC-like | ADC-like |
| 168 | GSM1213847 | ADC | ADC | ADC-like | ADC-like | ADC-like |
| **169** | **GSM1213848** | **ADC** | **SCC** | **SCC-like** | **SCC-like** | **SCC-like** |
| 170 | GSM1213849 | ADC | ADC | SCC-like | ADC-like | ADC-like |

**Table S4. The histological classification of samples classified by pathological assessment, signature and cluster in the GSE58661**

| **No.** | **Sampleid** | **Pathological lable** | **Signature label** | **TOP 1000 cluster label** | **TOP 2000 cluster label** | **TOP 3000 cluster label** |
| --- | --- | --- | --- | --- | --- | --- |
| 1 | GSM1213669 | SCC | SCC | SCC-like | SCC-like | SCC-like |
| 2 | GSM1213670 | ADC | ADC | ADC-like | ADC-like | ADC-like |
| 3 | GSM1213672 | ADC | ADC | ADC-like | ADC-like | ADC-like |
| 4 | GSM1213673 | SCC | SCC | SCC-like | SCC-like | SCC-like |
| 5 | GSM1213674 | ADC | ADC | ADC-like | ADC-like | ADC-like |
| 6 | GSM1213675 | SCC | SCC | SCC-like | SCC-like | SCC-like |
| 7 | GSM1213676 | ADC | ADC | ADC-like | ADC-like | ADC-like |
| 8 | GSM1213679 | SCC | SCC | SCC-like | SCC-like | SCC-like |
| 9 | GSM1213680 | SCC | SCC | SCC-like | SCC-like | SCC-like |
| 10 | GSM1213681 | ADC | ADC | ADC-like | ADC-like | ADC-like |
| 11 | GSM1213682 | SCC | SCC | SCC-like | SCC-like | SCC-like |
| 12 | GSM1213683 | ADC | ADC | ADC-like | ADC-like | ADC-like |
| 13 | GSM1213684 | SCC | SCC | ADC-like | ADC-like | ADC-like |
| 14 | GSM1213685 | ADC | ADC | ADC-like | ADC-like | ADC-like |
| **15** | **GSM1213686** | **ADC** | **SCC** | **ADC-like** | **ADC-like** | **ADC-like** |
| 16 | GSM1213688 | SCC | SCC | SCC-like | SCC-like | SCC-like |
| 17 | GSM1213689 | SCC | SCC | SCC-like | SCC-like | SCC-like |
| 18 | GSM1213690 | ADC | ADC | ADC-like | ADC-like | ADC-like |
| 19 | GSM1213692 | ADC | ADC | ADC-like | ADC-like | ADC-like |
| 20 | GSM1213693 | ADC | ADC | ADC-like | ADC-like | ADC-like |
| 21 | GSM1213694 | ADC | ADC | ADC-like | ADC-like | ADC-like |
| 22 | GSM1213695 | SCC | SCC | SCC-like | SCC-like | SCC-like |
| 23 | GSM1213696 | SCC | SCC | ADC-like | ADC-like | ADC-like |
| 24 | GSM1213697 | ADC | ADC | ADC-like | ADC-like | ADC-like |
| **25** | **GSM1213698** | **SCC** | **ADC** | **ADC-like** | **ADC-like** | **ADC-like** |
| 26 | GSM1213699 | ADC | ADC | ADC-like | ADC-like | ADC-like |
| **27** | **GSM1213700** | **SCC** | **ADC** | **ADC-like** | **ADC-like** | **ADC-like** |
| 28 | GSM1213701 | ADC | ADC | ADC-like | ADC-like | ADC-like |
| 29 | GSM1213702 | SCC | SCC | SCC-like | ADC-like | ADC-like |
| 30 | GSM1213703 | ADC | ADC | ADC-like | ADC-like | ADC-like |
| 31 | GSM1213704 | ADC | ADC | ADC-like | ADC-like | ADC-like |
| 32 | GSM1213705 | SCC | SCC | SCC-like | SCC-like | SCC-like |
| 33 | GSM1213706 | SCC | SCC | SCC-like | SCC-like | SCC-like |
| 34 | GSM1213707 | ADC | ADC | ADC-like | ADC-like | ADC-like |
| 35 | GSM1213708 | ADC | ADC | ADC-like | ADC-like | ADC-like |
| **36** | **GSM1213709** | **ADC** | **SCC** | **ADC-like** | **ADC-like** | **ADC-like** |
| 37 | GSM1213710 | ADC | ADC | ADC-like | ADC-like | ADC-like |
| 38 | GSM1213711 | ADC | ADC | ADC-like | ADC-like | ADC-like |
| 39 | GSM1213712 | ADC | ADC | ADC-like | ADC-like | ADC-like |
| 40 | GSM1213714 | ADC | ADC | ADC-like | ADC-like | ADC-like |
| 41 | GSM1213715 | SCC | SCC | SCC-like | SCC-like | SCC-like |
| 42 | GSM1213716 | ADC | ADC | ADC-like | ADC-like | ADC-like |
| 43 | GSM1213717 | SCC | SCC | SCC-like | SCC-like | SCC-like |
| 44 | GSM1213718 | SCC | SCC | SCC-like | SCC-like | SCC-like |
| 45 | GSM1213719 | ADC | ADC | ADC-like | ADC-like | ADC-like |
| 46 | GSM1213720 | ADC | ADC | ADC-like | ADC-like | ADC-like |
| **47** | **GSM1213721** | **SCC** | **ADC** | **ADC-like** | **ADC-like** | **ADC-like** |
| 48 | GSM1213722 | SCC | SCC | SCC-like | SCC-like | SCC-like |
| 49 | GSM1213723 | SCC | SCC | SCC-like | SCC-like | SCC-like |
| 50 | GSM1213724 | ADC | ADC | ADC-like | ADC-like | ADC-like |
| 51 | GSM1213725 | SCC | SCC | SCC-like | SCC-like | SCC-like |
| 52 | GSM1213729 | SCC | SCC | SCC-like | SCC-like | SCC-like |
| 53 | GSM1213730 | ADC | ADC | ADC-like | ADC-like | ADC-like |
| 54 | GSM1213731 | SCC | SCC | SCC-like | SCC-like | SCC-like |
| 55 | GSM1213732 | SCC | SCC | SCC-like | SCC-like | SCC-like |
| 56 | GSM1213733 | SCC | SCC | SCC-like | SCC-like | SCC-like |
| 57 | GSM1213734 | ADC | ADC | ADC-like | ADC-like | ADC-like |
| 58 | GSM1213736 | SCC | SCC | SCC-like | SCC-like | SCC-like |
| 59 | GSM1213737 | ADC | ADC | ADC-like | ADC-like | ADC-like |
| 60 | GSM1213738 | ADC | ADC | ADC-like | ADC-like | ADC-like |
| 61 | GSM1213739 | ADC | ADC | ADC-like | ADC-like | ADC-like |
| 62 | GSM1213740 | ADC | ADC | ADC-like | ADC-like | ADC-like |
| 63 | GSM1213741 | SCC | SCC | SCC-like | SCC-like | SCC-like |
| 64 | GSM1213742 | ADC | ADC | ADC-like | ADC-like | ADC-like |
| 65 | GSM1213743 | ADC | ADC | ADC-like | ADC-like | ADC-like |
| 66 | GSM1213744 | SCC | SCC | SCC-like | SCC-like | SCC-like |
| 67 | GSM1213745 | SCC | SCC | SCC-like | SCC-like | SCC-like |
| 68 | GSM1213746 | SCC | SCC | SCC-like | SCC-like | SCC-like |
| 69 | GSM1213747 | SCC | SCC | SCC-like | ADC-like | SCC-like |
| 70 | GSM1213748 | ADC | ADC | ADC-like | ADC-like | ADC-like |
| 71 | GSM1213749 | ADC | ADC | ADC-like | ADC-like | ADC-like |
| 72 | GSM1213750 | ADC | ADC | ADC-like | ADC-like | ADC-like |
| 73 | GSM1213751 | ADC | ADC | ADC-like | ADC-like | ADC-like |
| 74 | GSM1213752 | ADC | ADC | ADC-like | ADC-like | ADC-like |
| 75 | GSM1213753 | ADC | ADC | ADC-like | ADC-like | ADC-like |
| 76 | GSM1213754 | SCC | SCC | SCC-like | SCC-like | SCC-like |
| 77 | GSM1213755 | SCC | SCC | SCC-like | SCC-like | SCC-like |
| **78** | **GSM1213757** | **SCC** | **ADC** | **ADC-like** | **ADC-like** | **ADC-like** |

**
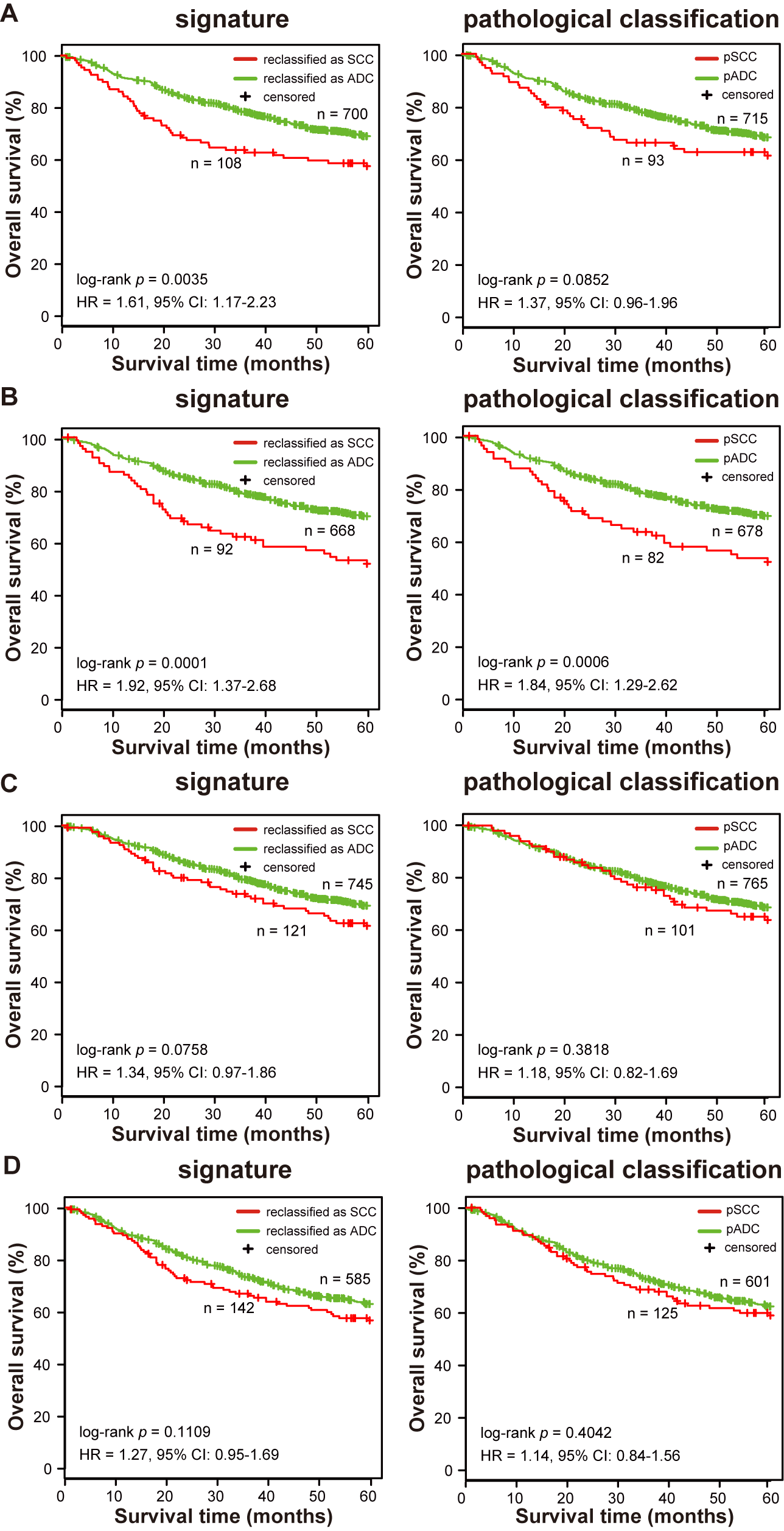

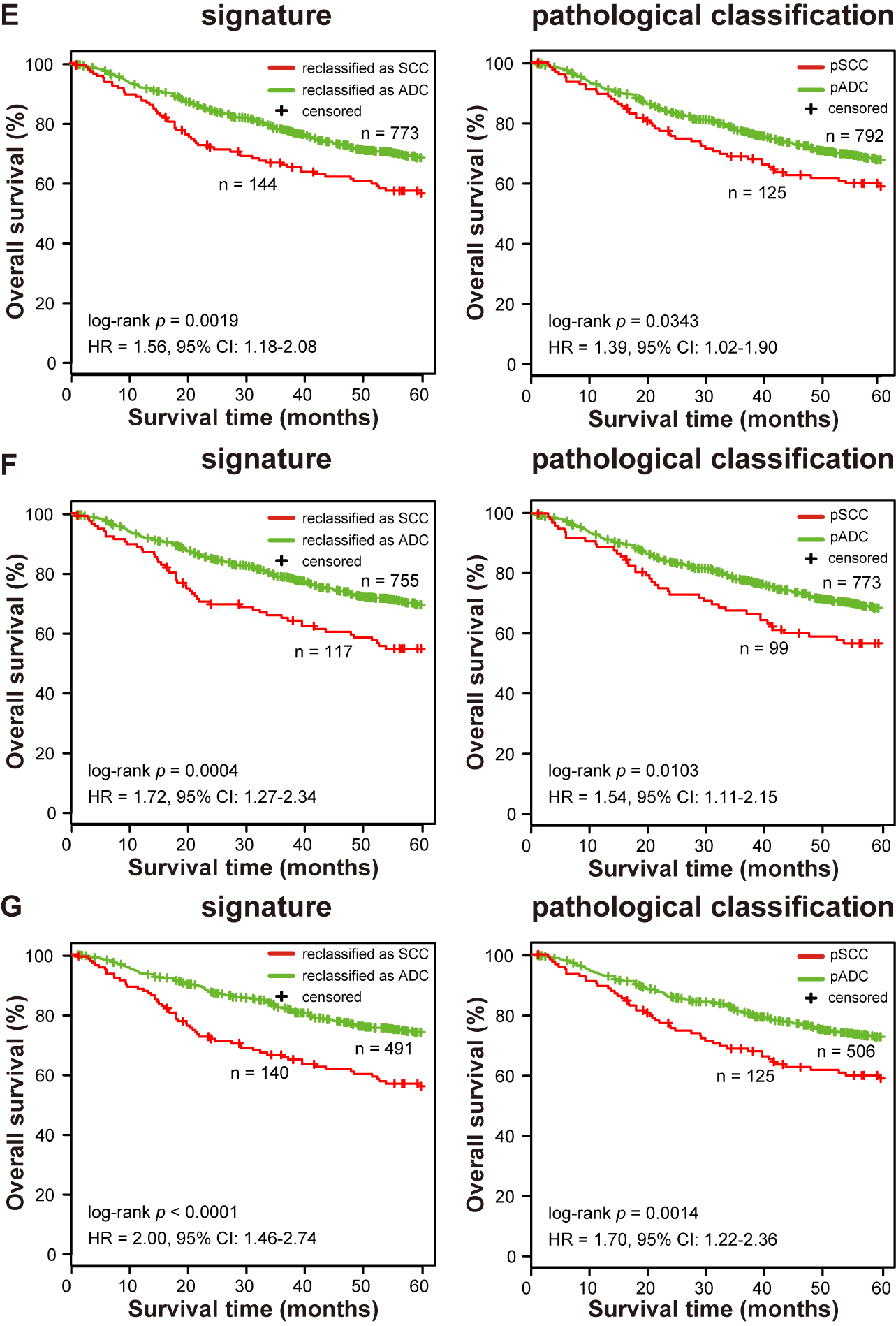
**

**Figure S1. Kaplan-Meier curves of OS respectively for the SCC and ADC groups reclassified by the signature and original pathological assessment in the data integrated by 6 datasets.** A-G were the survival results after excluding one dataset, respectively. The order of exclusion dataset was according to the integrated data in Table 2.

**
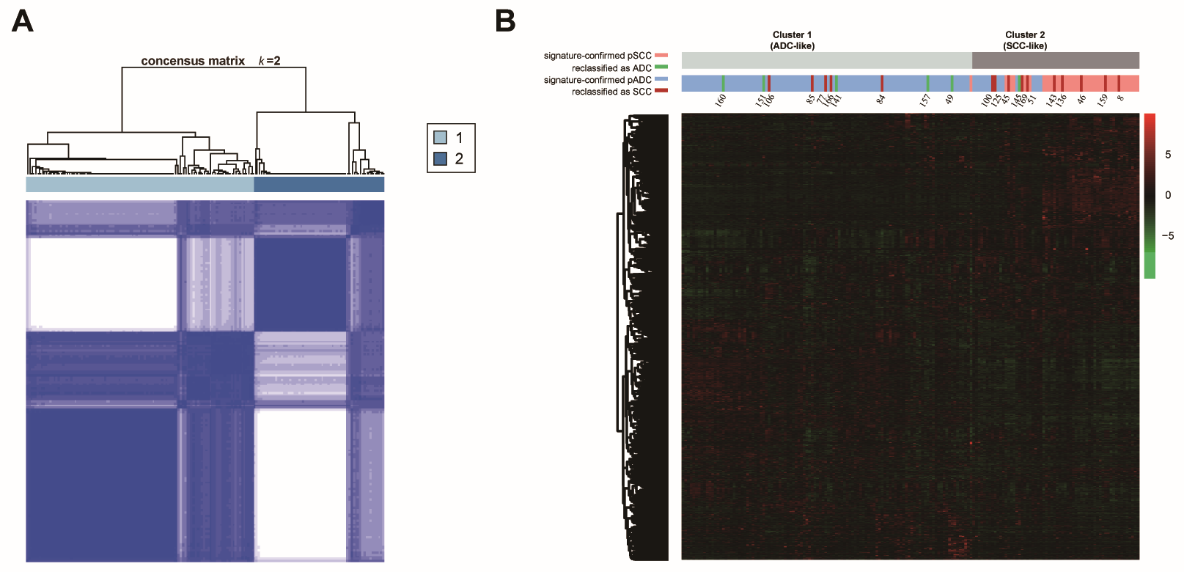
**

**Figure S2.** **The consensus clustering of the samples based on top 1000 most variable genes in the GSE50081 dataset.** (A) Consensus clustering heatmap of the 170 samples based on the top 1000 most variable genes. (B) Heatmap of samples ordered with the clustering result based on the top 1000 most variable genes. The 1000 most variable genes are ordered by hierarchical clustering to demonstrate their association with each cluster. The pSCC and pADC represent pathologically-determined SCC and pathologically-determined ADC, respectively. Cluster 1 and Cluster 2 include the most of signature-confirmed pADC samples and the most of signature-confirmed pSCC samples, which are denoted as ADC-like group and SCC-like group, respectively. The ID number of reclassified samples by the signature are displayed. The classifications of the samples are displayed in detail in Table S3.

**
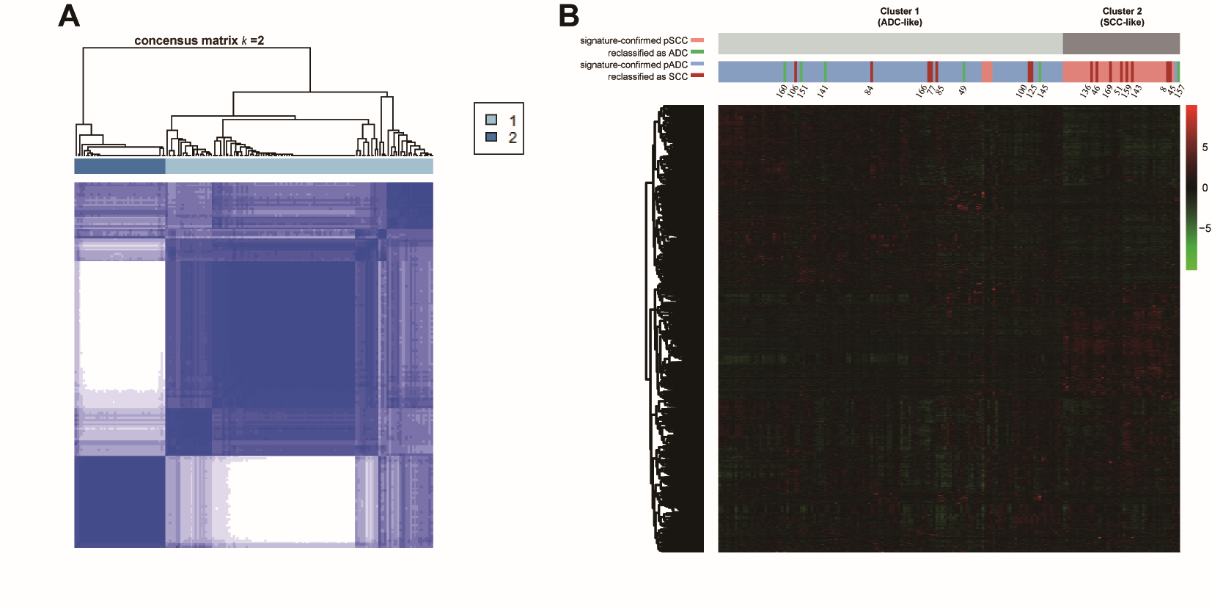
**

**Figure S3. The consensus clustering of the samples based on top 2000 most variable genes in the GSE50081 dataset.** (A) Consensus clustering heatmap of the 170 samples based on the top 1000 most variable genes. (B) Heatmap of samples ordered with the clustering result based on the top 2000 most variable genes. The 2000 most variable genes are ordered by hierarchical clustering to demonstrate their association with each cluster. The ID number of reclassified samples by the signature are displayed. The classifications of the samples are displayed in detail in Table S3.

**
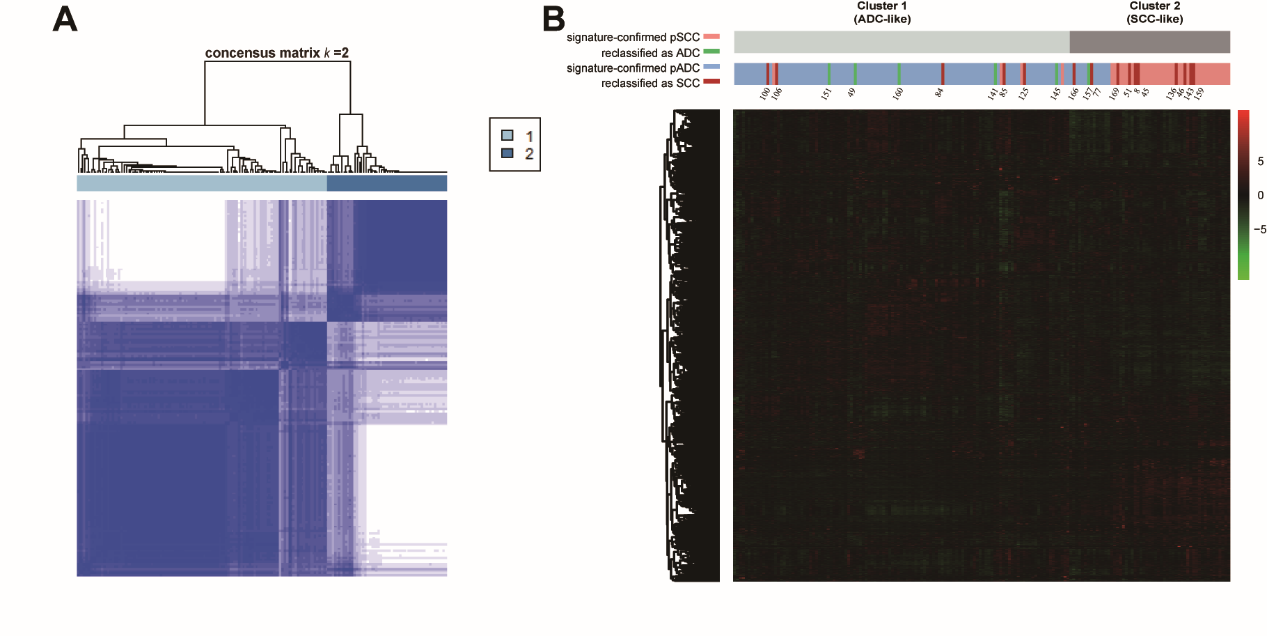
**

**Figure S4. The consensus clustering of the samples based on top 3000 most variable genes in the GSE50081 dataset**. (A) Consensus clustering heatmap of the 170 samples based on the top 1000 most variable genes. (B) Heatmap of samples ordered with the clustering result based on the top 3000 most variable genes. The 3000 most variable genes are ordered by hierarchical clustering to demonstrate their association with each cluster. The ID number of reclassified samples by the signature are displayed. The classifications of the samples are displayed in detail in Table S3.


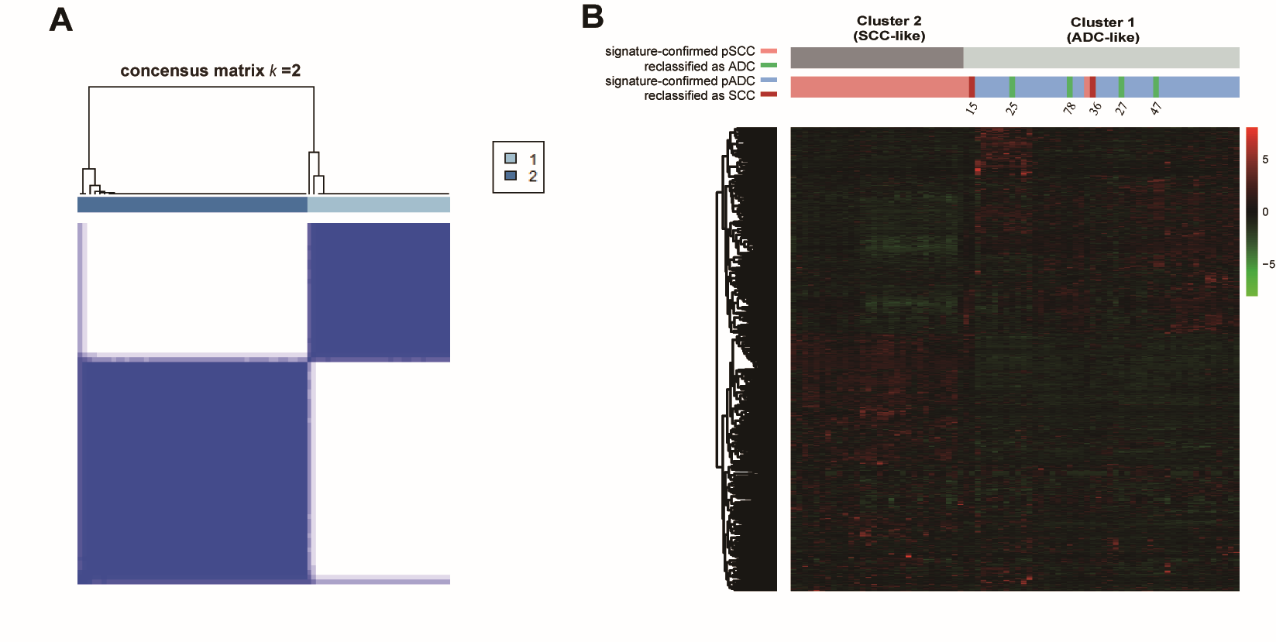


**Figure S5. The consensus clustering of the samples based on top 1000 most variable genes in the GSE58661 dataset for small biopsy specimens.** (A) Consensus clustering heatmap of the 78 samples based on the top 1000 most variable genes. (B) Heatmap of samples ordered with the clustering result based on the top 1000 most variable genes. The ID number of reclassified samples by the signature are displayed. The classifications of the samples are displayed in detail in Table S4.


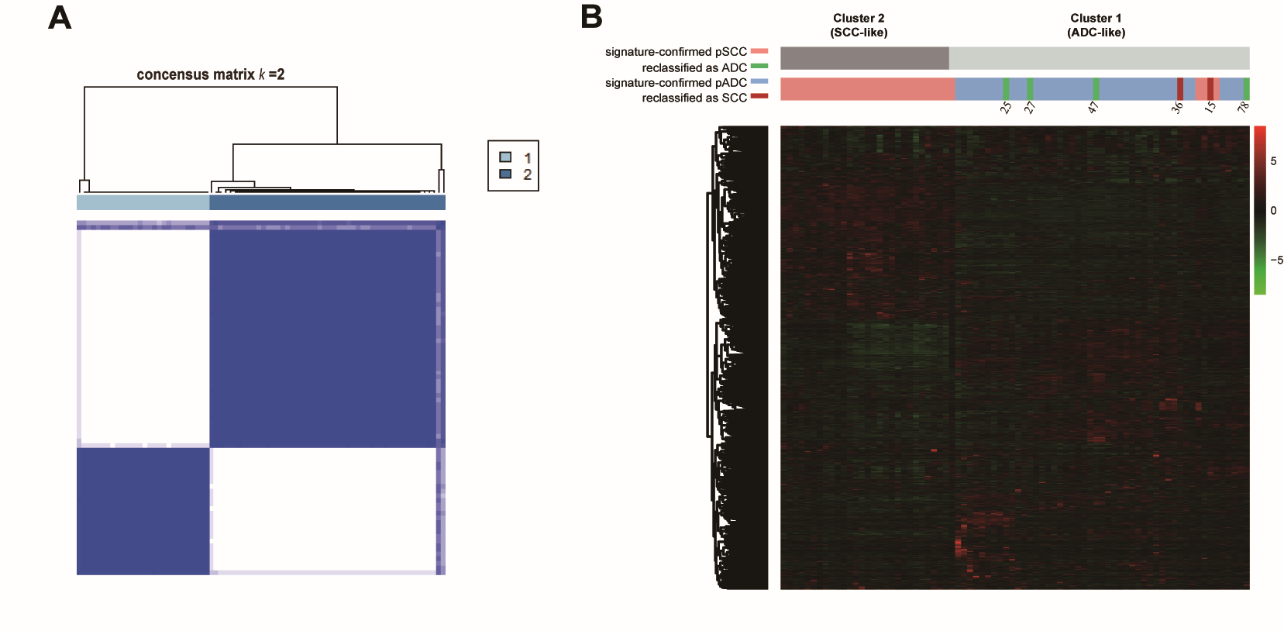


**Figure S6. The consensus clustering of the samples based on top 2000 most variable genes in the GSE58661 dataset for small biopsy specimens.** (A) Consensus clustering heatmap of the 78 samples based on the top 2000 most variable genes. (B) Heatmap of samples ordered with the clustering result based on the top 2000 most variable genes. The classifications of the samples are displayed in detail in Table S4.


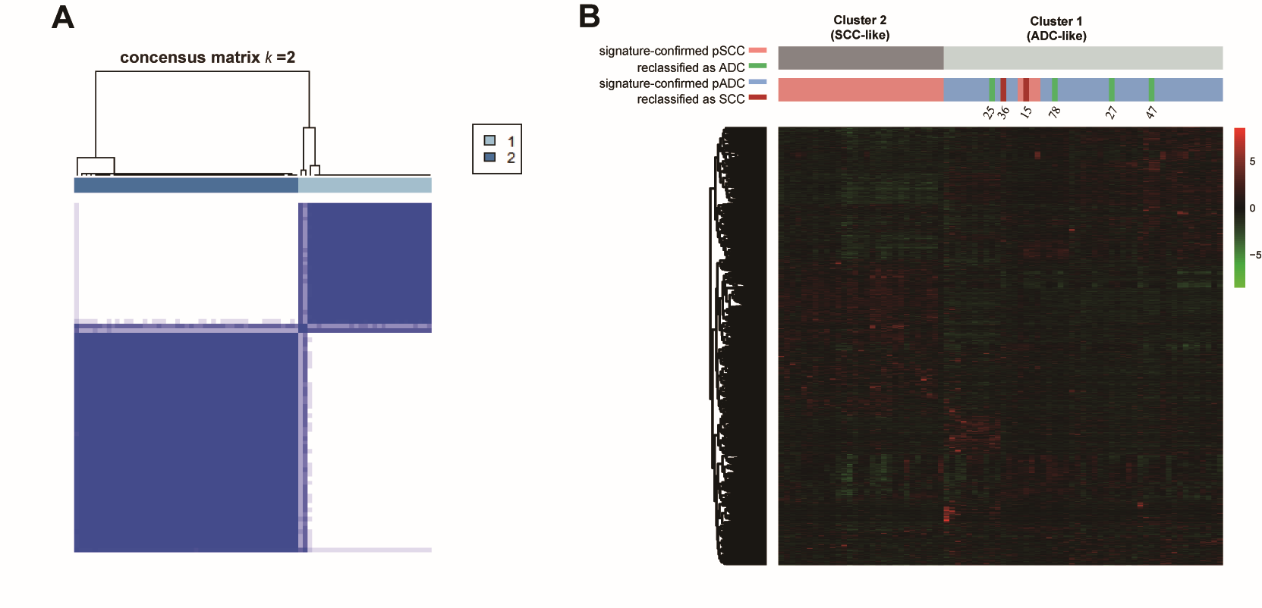


**Figure S7. The consensus clustering of the samples based on top 3000 most variable genes in the GSE58661 dataset for small biopsy specimens.** (A) Consensus clustering heatmap of the 78 samples based on the top 3000 most variable genes. (B) Heatmap of samples ordered with the clustering result based on the top 3000 most variable genes. The classifications of the samples are displayed in detail in Table S4.


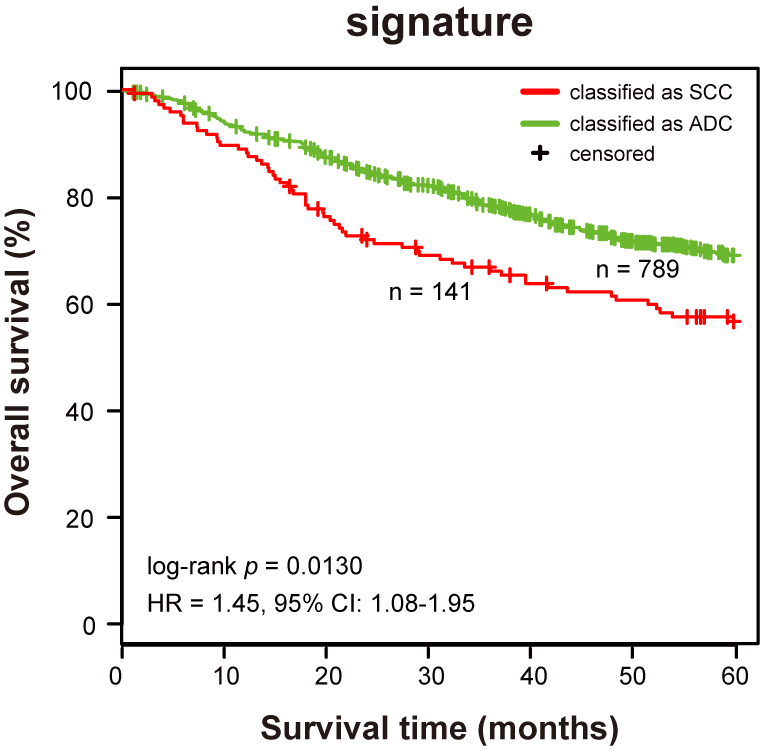


**Figure S8. The Kaplan-Meier curves of overall survival respectively for the ADC and SCC groups of patients treated with curative surgery resection only.** The SCC patients reclassified by the 42-signature had significantly shorter OS than the ADC, which was less significant than the OS difference between the two groups reclassified by our signature.
